# Enhanced Photosensitivity and Surfactant Resistance in Nanopolymersome Membranes as a Function of Gold Nanoparticle Incorporation

**DOI:** 10.1021/acsaom.4c00503

**Published:** 2025-02-11

**Authors:** Regina L. Salzer, Ajay N. Shah, Cory J. Trout, Abby R. Robinson, Sujay Ratna, Sean M. O’Malley, Julianne C. Griepenburg

**Affiliations:** † Center for Computational and Integrative Biology, 15636 Rutgers University-Camden, Camden, New Jersey 08102, United States; ‡ Department of Physics, Rutgers University-Camden, Camden, New Jersey 08102, United States; § Department of Chemistry, Rutgers University-Camden, Camden, New Jersey 08102, United States

**Keywords:** polymersomes, plasmonic, surfactant, light-responsive, pulsed laser, vesicles, gold nanoparticles

## Abstract

Polymersomes hold great promise as carrier vesicles for the encapsulation and delivery of cargo. Self-assembly of amphiphilic diblock copolymers into spherical vesicles result in two compartments for encapsulation: an aqueous lumen and a bilayer membrane. Herein, it is demonstrated that dodecanethiol-functionalized gold nanoparticles (AuNPs) can be loaded within the hydrophobic region of the bilayer membrane of polybutadiene-*b*-poly­(ethylene oxide) nanopolymersomes as photosensitizers. This is shown to render vesicles responsive to picosecond pulsed irradiation at a wavelength congruent with the localized surface plasmon resonance of the gold nanoparticles. Membrane disruption is demonstrated to scale with laser pulse energy and shows a strong enhancement with nanoparticle incorporation even at the low end of the concentration range. Nanoparticle concentration is also shown to increase polymersome stability in the presence of nonionic surfactants such as polysorbate 20 and ionic surfactants such as sodium dodecyl sulfate. In addition, a comparative analysis is performed between dynamic light scattering and cryo-transmission electron microscopy vesicle size results whereby an inference is made between sample composition and analytical method.

## Graphical abstract

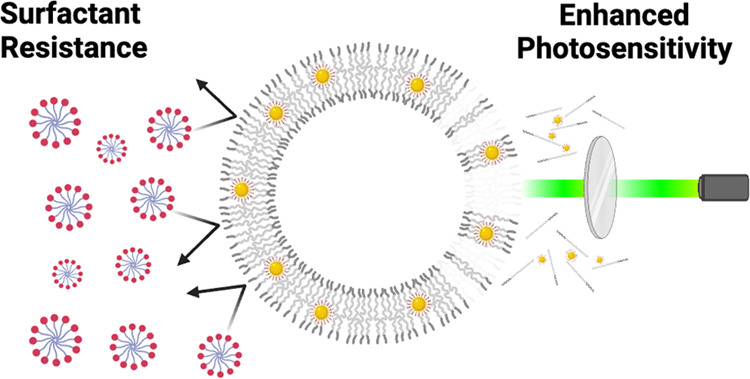

## Introduction

Carrier vesicles are an area of interest for use in various systems to encapsulate and deliver cargo. Liposomes, biomimetic vesicles with a phospholipid bilayer, have historically been studied for use as carrier vesicles with some liposomal drug delivery systems receiving approval from the United States Food and Drug Administration for use in clinical settings.^1,2^ More recently, synthetic polymeric vesicles, known, as polymersomes, have been regarded as a promising alternative as they offer favorable properties such as increased membrane thickness and stability.^3,4^ These carrier vesicles, comprised of amphiphilic block copolymers, can self-assemble into vesicles with an aqueous lumen and a bilayer membrane.^5^ Diblock copolymers in particular are commonly used to form polymersomes due to their similarities to biologically prevalent phospholipids found in cell membranes, both consisting of a hydrophobic and hydrophilic region. The synthetic nature of polymersomes allows for high levels of tunability such as altering membrane thicknesses between a broad range of 5–50 nm by changing the hydrophobic block length.^6^ The thick polymer bilayer also allows for the dual encapsulation of both hydrophobic and hydrophilic cargo in the bilayer membrane and the aqueous lumen, respectively.^6,7^ A wide range of commercially available synthetic block copolymers allows for tunability such as inherent responsiveness or robustness against internal and external stimuli.^8^ A vesicle system that triggers release upon external stimuli is particularly advantageous as it may offer superior control in terms of spatial and temporal resolution.

Several stimuli have been studied for releasing encapsulated cargo from polymersomes, including pH, temperature, and ultrasound waves, among others.^9–16^ However, these cargo release mechanisms offer relatively low spatial resolution and cannot be applied in a highly controlled manner due to their reliance on broader environmental changes or mechanical disruptions. Light as a stimulus offers a spatiotemporally precise release mechanism, triggering a thermomechanical response in the vesicle membrane that in turn prompts the release of the encapsulated cargo, a technique that has been shown to successfully release cargo in liposomal carrier systems.^17–19^ Lasers are particularly well-suited for this application, either as continuous wave (CW) or pulsed irradiation. Traditional mechanisms of photomediated cargo release often require extended irradiation times with a CW laser, but prolonged exposure can lead to environmental heating and damage of thermosensitive systems.^20^ The ability to reduce irradiation times and increase temporal release characteristics have led to the exploration of pulsed lasers as an alternative irradiation source.^21^ The short pulse duration associated with pulsed lasers can reduce the irradiation time required for cargo release, thus decreasing off-target heating.^19,22–24^

While many diblock copolymers are not inherently responsive to light, the incorporation of photosensitizers enables a tunable system which can render polymersomes light-responsive for cargo release at a chosen wavelength. Several small molecule options exist for photosensitizers,^25–27^ however, the unique and tunable optical properties of gold nanoparticles (AuNPs) make them especially suitable for this application.^24,28–31^ Gold nanoparticles exhibit strong light absorption at specific wavelengths due to the localized surface plasmon resonance (LSPR), a phenomenon caused by the collective oscillation of conduction band electrons in response to incident light.^32,33^ The LSPR wavelength is highly tunable and can be shifted depending on AuNP size and shape, offering another layer of control over the characteristics of the photosensitive system.^34–36^ The methodology for integrating AuNPs into carrier vesicle systems varies, but success has been achieved by functionalizing AuNPs with hydrophobic ligands such as dodecanethiol (DDT) to allow for AuNP integration into the hydrophobic region of the vesicle bilayer.^37–41^ Polymersome systems have been successfully photosensitized in this manner and shown to reduce the energy required to rupture the vesicles in response to pulsed irradiation.^42^ Subsequent studies found that irradiating vesicles containing AuNPs with energies below the rupture threshold resulted in cargo release, suggesting membrane poration while maintaining overall vesicle integrity.^42^ This promising result, demonstrated on micron-scale polymersomes, suggests that AuNPs can be utilized as a highly effective photosensitizer in polymersome carrier vesicle delivery systems, allowing for cargo release via an external, biologically compatible light stimulus.

In order to gain full control over cargo release from light-responsive polymersomes, it is important that these vesicles are robust in harsh conditions, for example, in the presence of surfactants. Though polymer membranes are frequently more resistant to stimuli than lipid membranes, polymer membranes are still vulnerable to solubilization via low molecular weight surfactants.^43^ Surfactants are prevalent in several biological environments that may be systems of interest for potential drug delivery targets, most notably in the respiratory system where pulmonary surfactant is present in relatively high concentrations on lung alveoli.^44,45^ Therefore, surfactant resistance is an attractive property for carrier vesicles for future use in biomedical applications. Examining vesicle response to low molecular weight surfactants provides insight into polymer membrane behavior and the role of membrane organization and structure in preserving membrane integrity. There have been successful methods of developing surfactant-resistant polymersomes through various methods such as increasing membrane thickness, utilizing amine cross-linkers, or adding macromolecular chain transfer agents.^46–49^

As AuNPs have been demonstrated to successfully photosensitize carrier vesicle systems, a more comprehensive study on the impact of membrane-incorporated AuNPs is warranted. Herein, we investigate the effects of changing the concentration of dodecanethiol-AuNPs (DDT-AuNPs) incorporated within nanoscale polymersomes during the self-assembly process. Samples were analyzed for photosensitivity and stability, and the results indicate that DDT-AuNP concentration not only changes photosensitivity, but also vesicle properties such as size, membrane fill percentage, and interestingly, robustness against commonly used surfactants. This light-responsive carrier has the potential to enhance applications that require the ability to gain control over cargo release, for example, drug delivery and microreactors.

## Methods and Materials

### Preparation of the Nanopolymersomes

Nanopolymersomes were prepared from an amphiphilic diblock copolymer, polybutadiene-*b*-poly­(ethylene oxide) (PBD_33_-*b*-PEO_20_) (Figure SI-1) (Polymer Source, Montreal, QC, Canada) via a direct solvent injection method.^42,50^ Diblock copolymer was dissolved in tetrahydrofuran (THF, anhydrous, 99.9% inhibitor free), (Sigma-Aldrich, St. Louis, Missouri) at a concentration of 2 mg/mL. This organic solution was injected dropwise from a 18G blunt tip needle into aqueous buffer (Milli-Q DI H2O) at a 3:7 v/v ratio, while stirring with a magnetic stir bar. ImageJ analysis of cryo-TEM images of vesicles formed without AuNPs found an average polymersome membrane thickness of 11.39 nm (Figure SI-2), which falls within the range of polymer membrane thicknesses found in literature.^51^ This membrane thickness is compatible with the incorporation of small, 3–5 nm AuNPs within the membrane. Dodecanethiol-functionalized gold nanoparticles (3–5 nm particle size, 2% w/v in toluene, Sigma-Aldrich) were heat dried and resuspended in the polymer-THF solution at the different concentrations studied (0.0175%, 0.0350%, 0.0525%, 0.0700%, and 0.140% w/v, as indicated) prior to self-assembly. The suspension was placed in a 3 mL dialysis cassette with a 20 000 Da molecular weight cut off (Slide-a-lyzer, Thermo Fisher) and allowed to dialyze with gentle stirring in 2 L water at 5 °C for 12–24 h to remove any remaining organic solvent.

The size distribution of the nanopolymersomes was determined via dynamic light scattering (DLS) using a Malvern Zetasizer Nano ZS with a scattering angle at 173°. The material was set to polyethylene glycol 400 with an index of refraction of 1.467 and absorption coefficient of 0.001. The samples were prepared for DLS by diluting (∼33-fold) with DDI H_2_O into a polystyrene disposable cuvette (Sigma-Aldrich) to a total volume of 1 mL. This dilution was chosen to yield an appropriate scattered light count for accurate results, as low concentrations may yield weak signals and high concentration may encounter particle–particle interactions which alter scattering. For each data point, three individual samples were measured in triplicate, with each scan consisting of 12 runs per scan at 25 °C. The triplicate scans were averaged for each sample, and subsequently the distributions of the three samples were averaged. Samples with a distribution size (by number) in the range of 50 to 120 nm, with a polydispersity index (PDI) < 0.200 were chosen for this study. Samples that indicated micelles (<35 nm), larger aggregates (>150 nm), or high PDI (>0.250) were discarded.

### Nanopolymersome Cryo-Transmission Electron Microscopy

To facilitate imaging via cryogenic transmission electron microscopy (cryo-TEM), the nanopolymersome samples were concentrated using a vacuum concentrator (Eppendorf Vacufuge Plus) by 15-fold prior to vitrification. Vitrification was performed using a manual plunge freezer (EMS, model EMS-001, Hatfield, PA), where 5 μL of the sample was pipetted onto a lacey carbon covered copper TEM grid (400 mesh, Ted Pella, Redding, CA). Front side blotting was manually performed with filter paper, followed by immediate plunging into liquid ethane cryogen maintained at −180 °C. The grids were then transferred to a Gatan ELSA single-tilt cryo-holder prior to imaging in a JEOL 2100Plus transmission electron microscope equipped with a 4k Rio Camera (Gatan, Pleasanton, CA). Imaging was conducted at 200 keV at a magnification of 30,000×.

### Laser Parameters/Irradiation

Prior to irradiation, the vesicle samples were placed into a 500 μL quart cuvette (1 cm path, Sigma-Aldrich, St. Louis, Missouri). Sample irradiation was performed using a 25 ps Nd:YAG laser (Ekspla Model PL2241, Vilnius, Lithuania), operating at 532 nm with a repetition rate of 250 Hz. The incident beam was quasi-collimated using a 1000 mm plano-convex lens (Thorlabs Inc., Newton, NJ), and the cuvette was positioned at 65 cm from the lens such that the beam diameter visually spanned the width of the cuvette. Samples were irradiated for 5 min using the following pulse energies: 0.1, 0.5, 1.0, and 2.0 mJ. Spot size (radius) at the cuvette was 7.91 × 10^–4^ m, as measured using the knife edge method (Figure SI-3). Each irradiation was performed in triplicate, and triplicate DLS scans of 12 runs each were taken for each sample before and after irradiation. Caution must be taken when operating Class 4 lasers, including appropriate safety goggles and optical barriers.

### Surfactant Studies

For the surfactant studies, DLS was used to monitor the vesicle size in response to the addition of surfactants. The as-prepared nanopolymersome samples were diluted 33-fold and added to a disposable polystyrene cuvette (1 mL). A 200 mM stock solution of the surfactant was added incrementally to the cuvette prior to scanning in triplicate. Changes in the size distribution by number were recorded for rupture analysis. Surfactants used included Triton X-100 (Sigma-Aldrich, St. Louis, Missouri, quality >200), polysorbate 20 (Sigma-Aldrich, St. Louis, Missouri, quality >300), sodium dodecyl sulfate (Sigma-Aldrich, St. Louis, Missouri, > 99%), and sodium deoxycholate (Sigma-Aldrich, St. Louis, Missouri, >97%)

## Results and Discussion

### Polymersomes Loaded with Different Concentrations of DDT-AuNPs

The effect of concentration of DDT-AuNPs on the AuNP incorporation into the polymersome membrane was investigated with cryo-TEM whereby the size of the polymersomes and the percentage of the membrane filled by the nanoparticles was determined. As shown in Figure 1A, empty polymersomes display a distinct membrane and hollow core. Figure 1B–D represent images of polymersomes self-assembled in the presence of 0.0350, 0.0700, and 0.140% w/v DDT-AuNPs. Interestingly, the AuNPs appear to organize nonuniformly in the vesicle membrane (Figure 1). This preferential organization was seen in previous work, and can likely be attributed to ligand–ligand interactions causing 2D close packing of spheres.^52^ This is similar to that seen in self-assembled monolayers (SAMs) except here they are confined by the membrane. On-going studies regarding alkanethiol-functionalized AuNP organization in vesicle membranes are warranted to further examine this phenomenon and how the ligand plays a role in packing.^42,52,53^ While the membrane in these images cannot be visually distinguished from the core due to the high contrast of the AuNPs, the AuNPs adopt the spherical shape of the vesicle suggesting membrane incorporation. A minimum of 27 polymersomes were measured from each sample. The size distribution of the polymersomes displays a log-normal shape as shown in Supporting Information, Figure SI-5. Given the log-normal nature of the distributions, the geometric mean was used to characterize the samples. The polymersome size (Figure 2) remains relatively consistent with a mean diameter of approximately 50 nm across samples at 0%, 0.0350% w/v, and 0.0700% w/v AuNPs. However, samples prepared with a concentration of 0.140% w/v were larger at 58 nm. The fill percentage of AuNPs in the membrane was determined using a protocol based on selective area contrast measurements. The average brightness of a completely filled region (*F*) of a polymersome (top and bottom hemispheres of the membrane) along with a region of the polymersome that is completely empty (*E*) were measured. The average fill percent (*x*) can be determined by measuring the brightness of an entire vesicle (*y*) and calculating *x* using eq 1

1 $$y\left(x\right)=\frac{F-E}{100}x+E$$

The fill percentage of AuNPs was measured to be approximately 20% (±2.5%), 48% (±7.34%), and 52% (±9.20%) for polymersomes prepared with 0.035, 0.0700, and 0.140% w/v, respectively, reported in Figure 2. While increasing the AuNP concentration from 0.0350 to 0.0700% w/v resulted in an increase in fill percentage, this trend did not continue with further increase in AuNP concentration. This suggests that there is a maximum membrane volume percentage that can be occupied by the AuNPs. However, it should be noted that the 0.140% loaded vesicles were slightly greater in size allowing for the incorporation of additional nanoparticles. Despite increasing the AuNP concentration in the self-assembly process, fill percentages greater than 50% were not observed, indicating that the formation of vesicles with AuNPs above this threshold is unfavorable.

**Figure 1:**
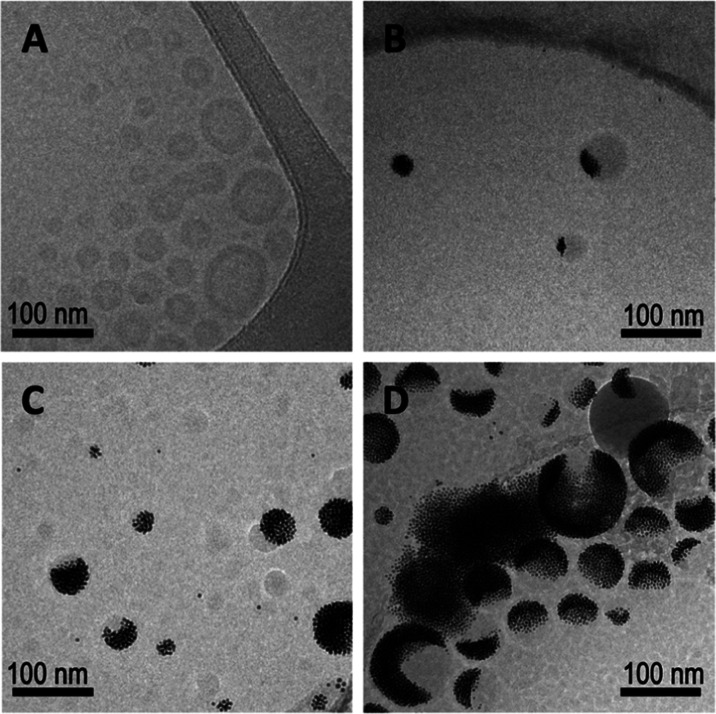
Representative cryo-TEM images of vesicles assembled with (A) 0% w/v, (B) 0.0350% w/v, (C) 0.0700% w/v, and (D) 0.140% w/v DDT-AuNPs in the membrane. A distinct membrane and core can be seen in the empty polymersomes in (A), but cannot be distinguished in (B–D) due to the high contrast of the AuNPs.

**Figure 2:**
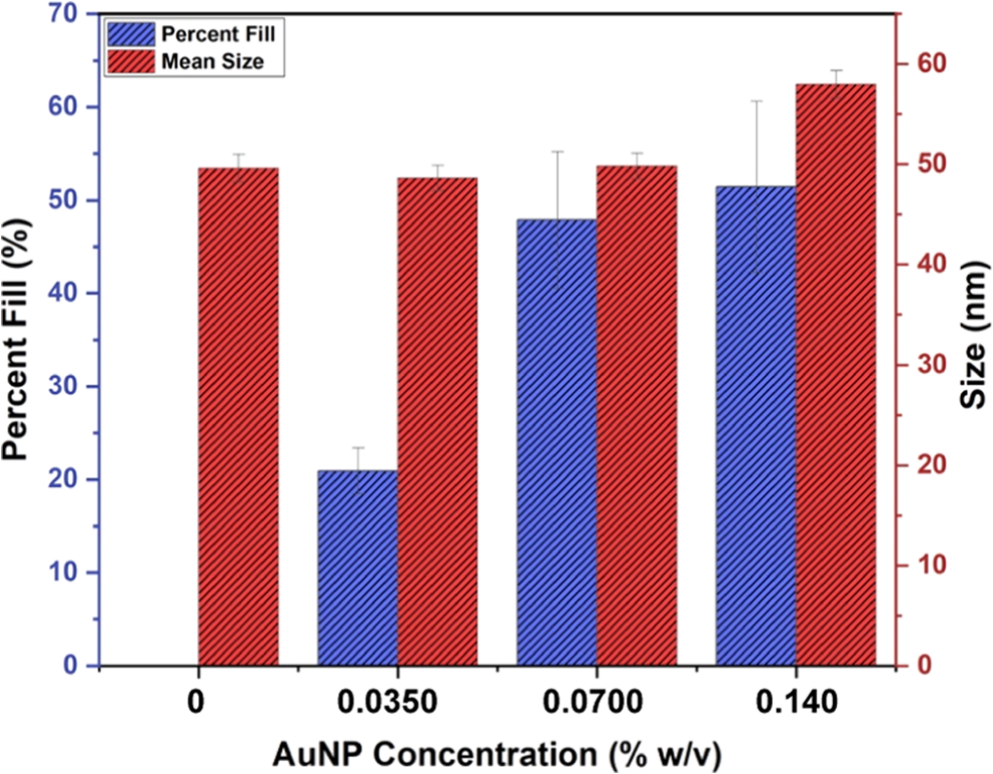
AuNP fill percentage within the membrane (blue) and mean vesicle size (red), as determined from cryo-TEM images by measuring intensity and size of individual vesicles with imageJ. Error bars represent the standard deviation from a minimum of 27 vesicles per sample.

### Irradiation of DDT-AuNP-Loaded Polymersomes

The photosensitivity of the polymersomes loaded with different concentrations of AuNPs was investigated using DLS before and after irradiation at different laser energies. The number distribution was used to characterize the samples due to its sensitivity to small particles (<30 nm), which is important for the detection of the formation of micelles upon polymersome disruption. The percent change in the median size and PDI before and after irradiation were calculated and averaged for irradiations of three identical samples. Monitoring relative changes in PDI following irradiation provides insight into the prevalence of different structures within a sample. The PDI is used to describe the width of the size distribution and calculated using the following equation where σ is the standard deviation and *Z*_D_ is the mean average diameter^54^

2 $$\mathrm{PDI}=\frac{\sigma^{2}}{Z_{D}^{2}}$$

With this metric, values between 0 and 0.2 are considered narrow distributions, between 0.2–0.4 are moderate, and greater than 0.4 are regarded as broad distributions. In this work, a significant increase in PDI after laser irradiation can be explained by disassembly of a portion of the vesicle population and assembly of smaller structures (e.g., micelles). These changes have the effect of increasing the standard deviation and reducing the mean size, both which contribute to an increasing PDI. Changes in the peak size and PDI after irradiation are indicative of membrane disruption, where a percent decrease in size of 65% or greater was taken to indicate the polymersomes have fully ruptured. This is consistent with the percent change associated with the minimum observed polymersome size (80 nm) transitioning to micelles of a maximum observed size (30 nm). Figure 3 shows that upon irradiation, the hydrodynamic diameter of the vesicle decreased (Figure 3A) while the PDI increased (Figure 3B); this trend correlated for both AuNP concentration and laser pulse energy. Polymersomes that were not loaded with AuNPs did not show any significant change in size or PDI over the range of laser energies. Polymersomes self-assembled with an AuNP concentration of 0.0350% w/v did not show a significant change in median size for laser energies of 0.5 mJ and lower. All of the samples loaded with AuNPs show significant changes in size (>65%) when irradiated with energies of 1.0 and 2.0 mJ. At the 0.1 mJ irradiation energy, only the sample containing the largest concentration (0.140% w/v) of AuNPs showed an increase in PDI. The 0.5, 1.0, and 2.0 mJ irradiations of samples containing any concentration of AuNPs resulted in noticeable increases in PDI. The greater dispersity of these samples indicates a larger range of sizes, strongly suggesting the presence of both micelles and bilayer vesicles.

**Figure 3:**
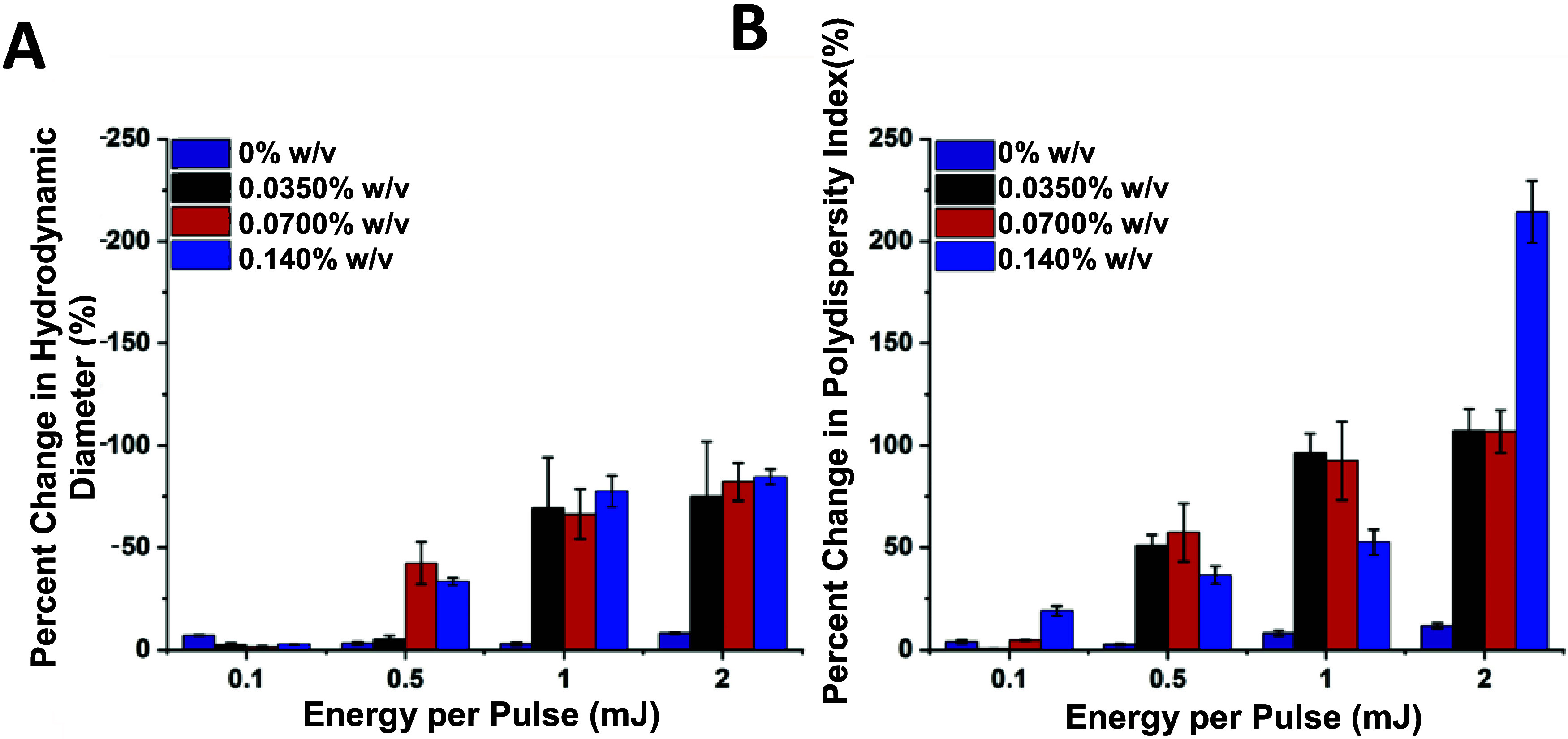
Dynamic light scattering data demonstrating the percent changes in (A) hydrodynamic diameter and (B) PDI for nanopolymersomes prepared with different concentrations of AuNPs after different energy irradiations. Error bars represent standard deviation from three individual samples.

The polymersome to micelle transition was investigated with cryo-TEM before and after irradiation for samples irradiated at the lowest and highest energies (0.1 and 2.0 mJ) across all concentrations of AuNPs. After irradiation, both polymersomes and micelles were observed, and the size distributions of both structural populations were measured (Figure SI-6). As shown in Figure 1A, intact polymersomes can be characterized by a spherical shape with organized AuNPs within the membrane. Any deviation from this appearance after irradiation can be attributed to membrane alterations and/or AuNP modification. Micelles are characterized by a significant decrease in size, not necessarily retaining a hollow appearance. Samples that were irradiated with the lower energy (0.1 mJ) did not undergo significant structural alterations, as only fully intact vesicles were observed after irradiation for all three loaded AuNP concentrations investigated. In all cases, the percent change in the size of the polymersomes after irradiation was less than 6.8%. However, the lack of AuNP organization within the membrane suggests AuNP modification in response to irradiation.^22^ In the case of 2.0 mJ irradiations, samples underwent significant changes, as shown in Figure 4A–C. Figure 4A represents a cryo-TEM image of a 0.0350% AuNP concentration sample irradiated with an energy of 2 mJ where intact polymersomes could still be observed. Notably, there are also changes to the size of the AuNPs. This can be attributed to thermal fusing which has been previously observed under picosecond irradiation.^7,55^ Increasing the concentration to 0.0700% results in a substantially lower number of intact polymersomes, with an increased number of micelles observed, as shown in Figure 4B. Finally, increasing to the highest concentration while keeping energy constant, resulted in scarce observation of intact polymersomes and/or micelles, as shown in Figure 4C. The changes in the distributions of polymersomes and micelles before and after irradiation are shown in Figure 5, emphasizing the role that concentration plays in the resulting structural alterations. Prior to irradiation, a monomodal distribution can be attributed to samples consisting of primarily polymersomes (Figure 5A–C). However, after irradiation (Figure 5D–F), polymersomes and micelles are represented by a bimodal distribution. In the case of high AuNP concentration, the counts are significantly diminished. This correlates well with Figure 4C where very few structures can be identified. It should be noted that samples devoid of AuNPs and irradiated with high energy did not undergo changes (Figure SI-2).

**Figure 4:**
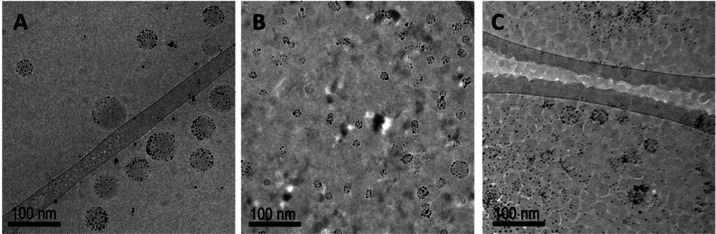
Representative cryo-TEM images of vesicles irradiated with 2 mJ energy assembled with (A) 0.0350% w/v, (B) 0.0700% w/v, and (C) 0.140% w/v DDT-AuNPs in the membrane. After irradiation, few intact polymersomes can be observed, especially in samples prepared with higher concentrations of AuNPs.

**Figure 5:**
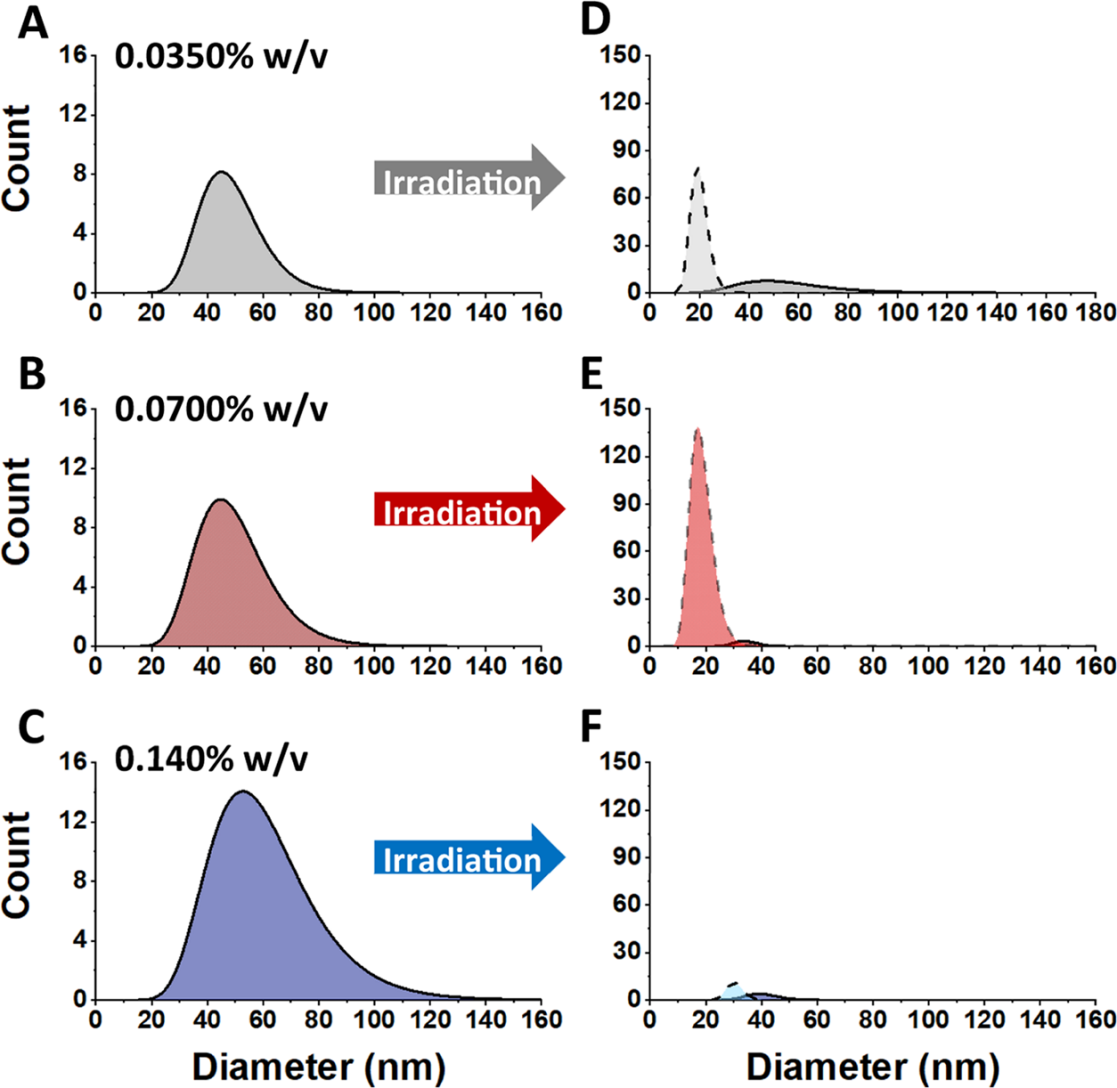
Distributions of polymersomes and micelles before (A–C) and after (D–F) irradiation with 2.0 mJ energy at the indicated concentrations. The micelle distributions are represented with dashed lines, while the vesicle distributions are indicated with solid lines. (A–C) show a single distribution indicating primarily vesicles prior to irradiation. After irradiation, bimodal distributions can be seen in (D-F), where the smaller sized particles can be attributed to micelles.

### Stability against Surfactants

The stability of the DDT-AuNP-loaded nanopolymersomes was investigated by subjecting the vesicles to various surfactants. Surfactants are defined as amphipathic molecules that can be either nonionic or ionic in nature, and these molecules are frequently used to disrupt bilayer membranes and solubilize membrane fractions.^56,57^ Polymersomes, like liposomes, have been demonstrated to be successfully solubilized using a variety of common surfactants such as sodium dodecyl sulfate (SDS), Triton X-100, and polysorbate 20.^58^ However, membrane properties have been demonstrated to impact the vesicle susceptibility to surfactants.^48,49^ As such, it can be hypothesized that constituents within the hydrophobic region of the membrane such as DDT-AuNPs could alter their response to surfactants.

Nanopolymersomes were self-assembled as described and monitored for changes in size via DLS in response to increasing concentrations of surfactant where the incremental increase in surfactant concentration was chosen based on vesicle response. Surfactants in two categories were investigated: (1) nonionic surfactants, including polysorbate 20 and Triton X-100, and (2) ionic surfactants, including ionic SDS and sodium deoxycholate. Figure 6 demonstrates how the vesicle size changes upon the addition of polysorbate 20 for three AuNP concentrations (0%, 0.0700%, and 0.140% w/v). Additional concentration data and distributions by intensity can be found in Figures SI-7 and SI-8. At all concentrations of gold, the samples start off as polymersomes (dark blue line), represented by a single peak greater than 50 nm. Polysorbate 20 was added to the sample volume until DLS indicated a single peak less than 35 nm demonstrating the sole presence of micelles (red line). This was taken to be the point of complete rupture.

**Figure 6:**
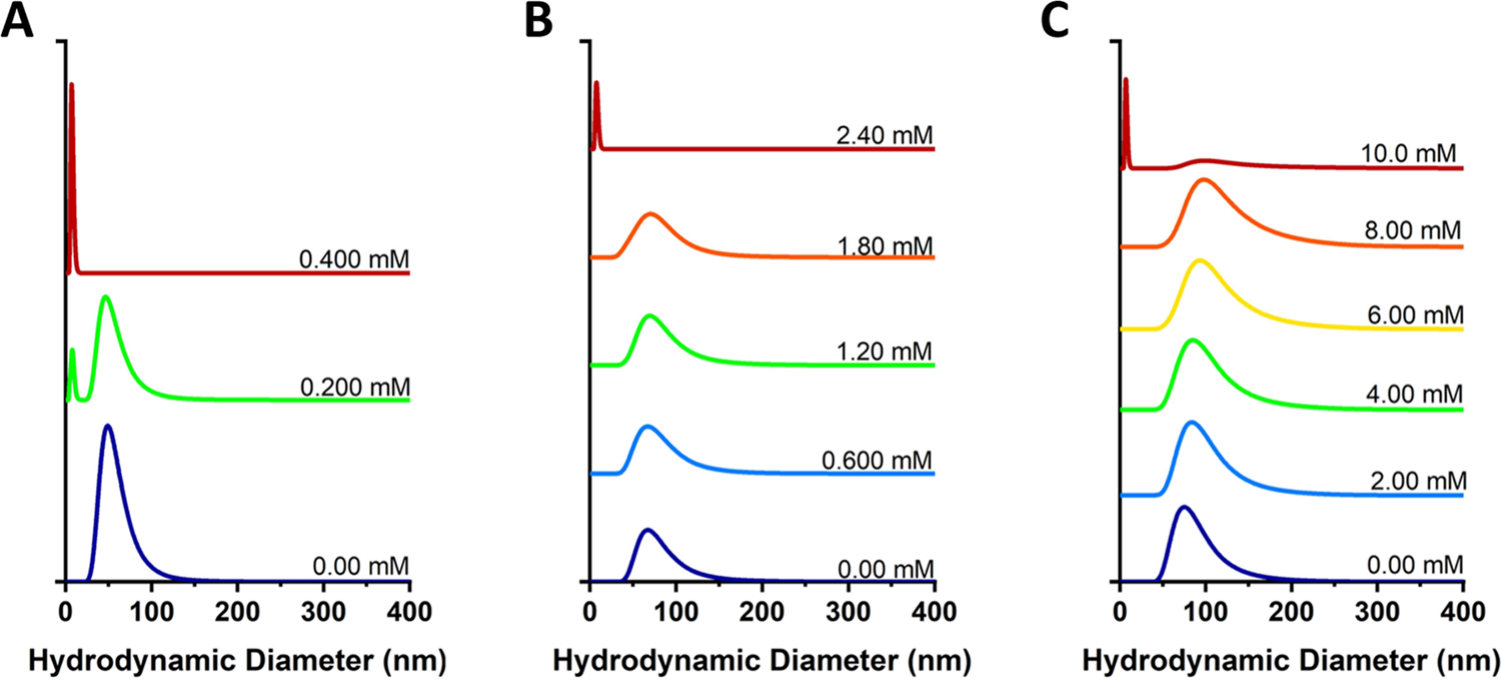
DLS distribution curves by number showing changes in hydrodynamic diameter in response to increasing concentrations of polysorbate 20 for nanopolymersomes self-assembled with (A) 0%, (B) 0.0700%, and (C) 0.140% w/v of DDT-AuNPs. The distributions are offset and stacked vertically to allow for the visualization of relative changes in hydrodynamic diameter.

Figure 7A shows the peak (mode) hydrodynamic diameter measured by DLS of vesicles as surfactant, polysorbate 20, was added. Nanopolymersomes without AuNPs (0% w/v) underwent a complete transition to micelles, i.e., rupture, with 0.4 mM polysorbate 20. Conversely, a concentration of 10.0 mM polysorbate 20 was required to completely rupture the vesicles with maximum gold loading (0.140% w/v), demonstrating a 25-fold increase in surfactant required for rupture. Further analysis of sample polydispersity (Figure 7B) reveals that higher gold concentrations aided in maintaining the monodispersity of the sample as surfactant concentration was increased. To probe whether this response was merely a result of a hydrophobic constituent contained within the membrane, similar experiments with both polysorbate 20 and SDS were conducted with Nile Red, a small molecule hydrophobic fluorophore, integrated into the membrane in lieu of AuNPs; no changes in surfactant response were detected as shown in Figures SI-9 and SI-10. This procedure was also performed with a second nonionic surfactant, Triton X-100. As shown as in Figures SI-11, SI-12, and SI-13, size changes follow a similar trend to that observed with polysorbate 20, whereby it requires 100x more Triton X-100 to induce the polymersome transition to micelles for vesicles assembled with the highest concentration of AuNPs.

**Figure 7:**
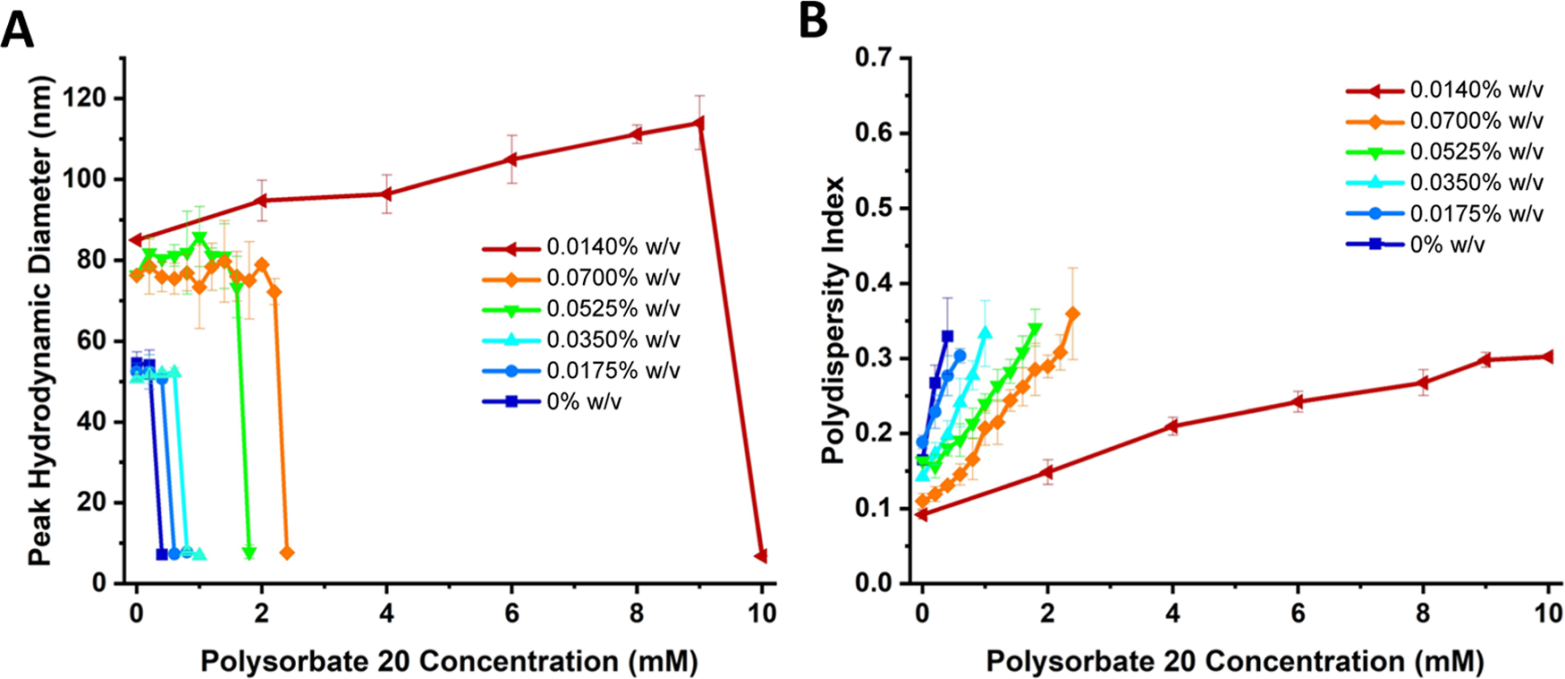
(A) Peak (mode) hydrodynamic diameter of polymersomes as a function of polysorbate 20 concentration. (B) PDI as a function of polysorbate 20 concentration. All measurements were performed in triplicate with error bars representing the standard deviation of the mean diameter (A) and PDI (B).

A similar methodology was used to study ionic surfactants. Figure 8 demonstrates changes in the average hydrodynamic diameter in response to ionic SDS addition for vesicles assembled with increasing amounts of AuNPs (see Figures SI-14 and SI-15 for additional AuNP concentrations and distributions by intensity). Intriguingly, vesicles assembled with AuNPs still did not undergo rupture at all and the size response to SDS differed from that seen with nonionic surfactants. It can be noted in Figure 9 that the addition of the ionic surfactant led to substantial vesicle size increases in samples prepared with AuNPs, up to 50%. However, no significant correlation was apparent between the increase in size and AuNP concentration. Figure 9A shows that vesicles containing AuNPs resisted a transition to micelles at high concentrations of SDS while samples self-assembled without AuNPs readily ruptured at a concentration of approximately 2.5 times the critical micelle concentration (CMC) of SDS (∼8 mM). Furthermore, Figure 9B demonstrates that vesicles formed in the presence of AuNPs maintain stable PDI values, with higher concentrations of gold (0.140% w/v) resulting in the lowest polydispersity. Vesicles assembled without AuNPs, which do undergo rupture in response to SDS addition, demonstrate a significant increase in PDI. As shown in eq 2, this significant increase in PDI can be attributed to an increase in standard deviation (σ) due to disrupted vesicular structures and a decrease in size (*Z*_*D*_) from the presence of micelles, both which contribute to increased PDI. This experiment was repeated was a second ionic surfactant, sodium deoxycholate, where the same trends were observed (Figures SI-16, SI-17, and SI-18). To ascertain if this could be attributed to changes in osmotic pressure, a parallel experiment was conducted with sodium chloride, with concentrations reaching over 600 mM; no rupture or swelling was observed, even in vesicles devoid of AuNPs (Figures SI-19 and SI-20).

**Figure 8:**
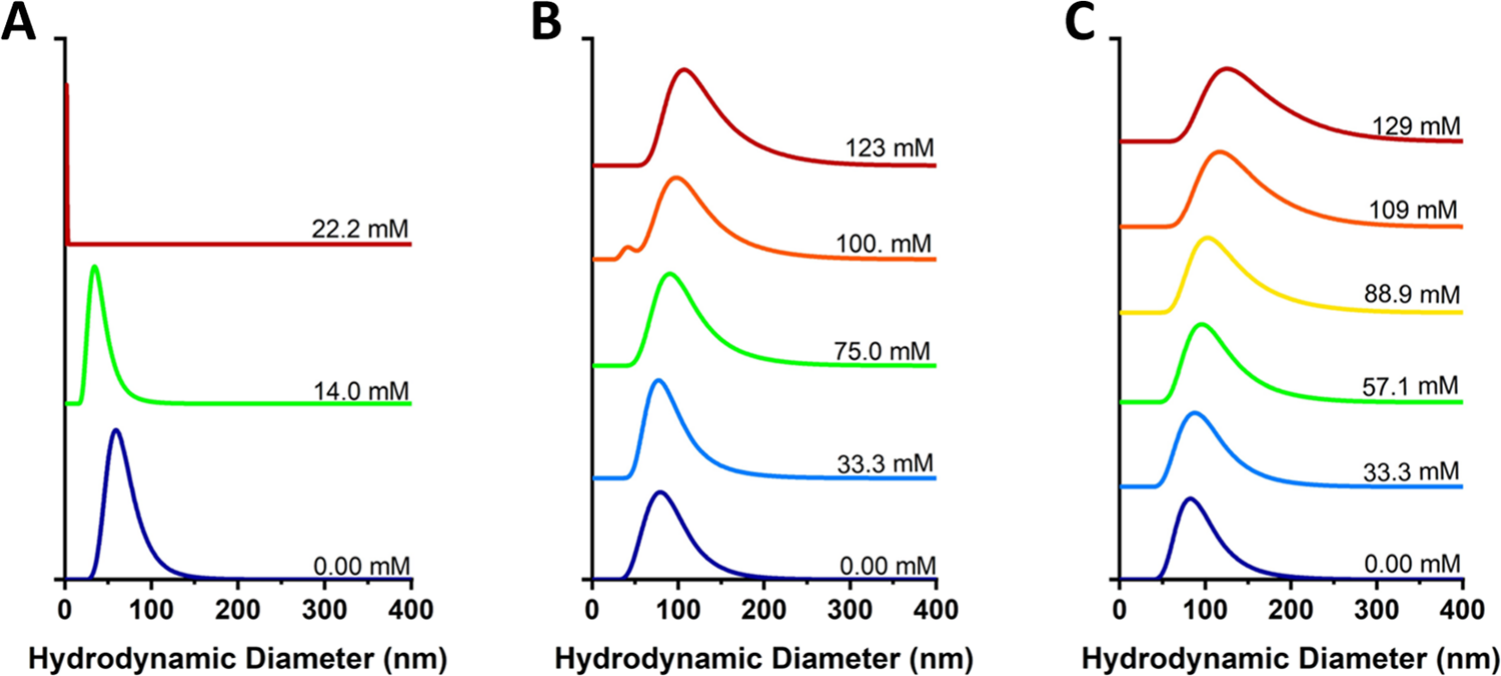
Nanopolymersome size distributions by number, measured via DLS, with increasing concentrations of SDS. (A) Vesicles containing 0% w/v AuNPs, (B) vesicles containing 0.0700% w/v AuNPs, and (C) vesicles containing 0.140% w/v AuNPs. The distributions are offset and stacked vertically to allow for the visualization of relative changes in hydrodynamic diameter.

**Figure 9:**
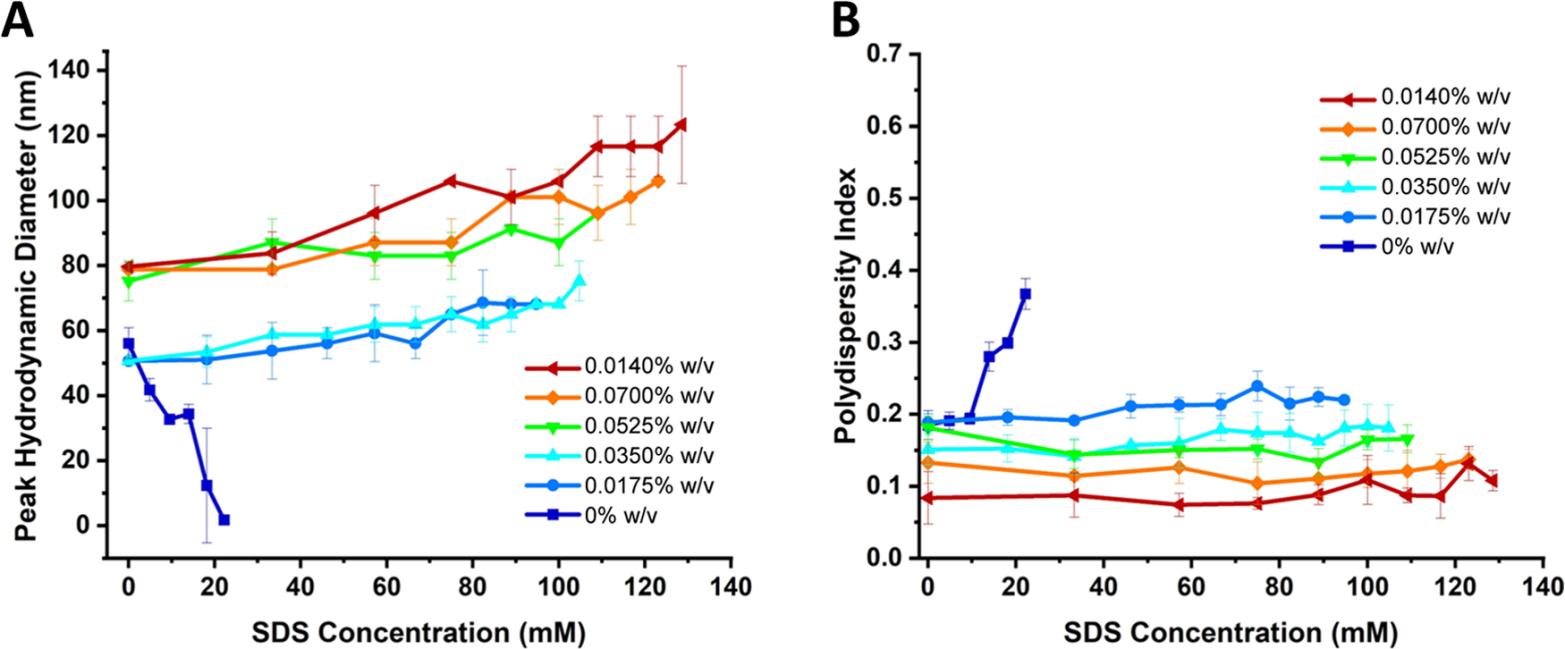
(A) Peak (mode) hydrodynamic diameter of polymersomes as a function of SDS concentration. (B) PDI as a function of SDS concentration. All measurements were performed in triplicate and error bars represent standard deviation of the mean diameter (A) and PDI (B).

The disruption of phospholipid bilayer membranes by surfactants is well documented in the literature, whereby surfactant molecules adsorb onto the exterior of the bilayer until the CMC is reached.^59,60^ Adding surfactant past the CMC leads to the intercalation of surfactant molecules into the exterior leaflet of the bilayer, causing asymmetric membrane stress.^61,62^ Nonionic surfactants are more likely to spontaneously move, or “flip-flop”, to the inner leaflet and rapidly reach equilibrium, while charged surfactants are less likely to flip-flop and accumulate almost exclusively in the exterior leaflet, often requiring a lower surfactant concentration for vesicle rupture.^63^ A sufficiently high concentration of surfactant is required to induce enough asymmetric stress such that the membrane begins to partition and bud off into mixed micelles composed of both membrane components and surfactant monomers.^62,64^ However, the exact method through which surfactants disrupt vesicle membranes may differ depending on the composition of the membrane.^65^ Liposomes are composed of phospholipids, most of which have relatively short lipid tails that form a bilayer only 3–5 nm thick, therefore producing a relatively thin hydrophobic membrane region and allowing for more surfactant trans-bilayer movement.^4,66–68^ Conversely, polymersomes have thicker membranes with a much larger hydrophobic membrane region, inhibiting the migration of surfactant molecules through the bilayer.^69^ Accordingly, increases in membrane thickness have been shown to change vesicle surfactant resistance, and the thickness of the hydrophobic region of the bilayer may alter the disruption of polymersome membranes by surfactants.^48^ Additionally, chemical cross-linking, such as amine cross-linkers have been shown to decrease susceptibility to surfactants.^49^

To further investigate the mechanism for surfactant resistance in AuNP-loaded polymersomes, a fluorescent probe known as Laurdan was utilized to detect changes in membrane fluidity in response to AuNP concentration. While Laurdan is more commonly used to investigate phase changes in liposomes, He and Tong demonstrated that it can be utilized to probe polymer membrane rigidity.^70^ Generalized polarization (GP) as defined by Parasassi et al. is the most commonly calculated parameter in literature when using Laurdan to probe the overall membrane order and can be calculated using the following equation where *I*_440_ is the fluorescence intensity of the sample at 440 nm and *I*_490_ is the fluorescence intensity at 490 nm^71^

3 $$\mathrm{GP}=\frac{I_{440}-I_{490}}{I_{440}+I_{490}}$$

The fluorescence spectrum used to calculate GP is shown in Figure SI-21. As shown in Figure SI-22, no considerable change in GP values calculated using eq 3 were found in response to AuNP membrane loading concentration; this suggests that changes in membrane rigidity do not play a role in the surfactant resistance demonstrated in this system. This result, combined with prior studies demonstrating surfactant resistance in response to chemical cross-linking, suggests that the AuNPs may be playing a similar role whereby the DDT-AuNP-polybutadiene interactions act as a scaffold to anchor the membrane, making it less susceptible to asymmetric stress. The DDT-AuNP-polybutadiene interactions may also impede the ability of surfactants to migrate into and through the bilayer membrane. Membrane thickness and composition has been shown to strongly influence vesicle resistance to surfactants and increased resistance to surfactants appears to rely on increasing molecular interactions within the hydrophobic region of the bilayer. Consequently, surfactants may act as a probe for identifying how other system inclusions such as membrane-embedded nanoparticles impact polymer membrane organization and robustness. Therefore, it is hypothesized that the DDT ligands on the functionalized AuNPs are interacting with the polybutadiene block of the copolymer, thus increasing intermolecular interactions within the hydrophobic region of the bilayer and leading to increased vesicle surfactant resistance. Interestingly, a significant increase in size as measured by DLS can be noted upon the addition of ionic surfactants, as shown in Figure 9. This is supported by the study by Chambon et al. which demonstrated an increase in cross-linked vesicle diameter as reported by DLS in response to ionic surfactants.^49^ This increase in size with surfactant concentration could be attributed to an increase in surfactant corona as well as a change in effective index of refraction.

### Comparison of Dynamic Light Scattering and Cryo-TEM for Polymersome Analysis

With the extensive size analysis performed via DLS and cryo-TEM within this study, a secondary outcome was the ability to compare the two techniques in terms of their utility and accuracy. Cryo-TEM has been long regarded as the gold standard for imaging vesicles, however, DLS is often employed due to its efficiency and ease of use.^72^ Interpretation of DLS data is often challenging as there are multiple analytical models that one can choose from (e.g., size distribution by number or intensity, as defined by Malvern Panalytical, Westborough, MA), each providing a different weighted distribution. Analytical model selection is further confounded by complex samples, such as those demonstrated in this work. Table 1 shows a comparison of sizes determined by cryo-TEM and DLS distribution by number and intensity. Size distribution by number more closely agrees with the mean size of polymersomes determined by the cryo-TEM images. The larger reported value for DLS size distribution by number is likely due to the fact that DLS measures the hydrodynamic size of particles in solution. This indicates that the number distribution is fairly accurate for the homogeneous solutions such as those present prior to irradiation, however, the model fails to resolve samples consisting of two populations (i.e., polymersomes and micelles) after irradiation. The intensity distribution, however, was able to resolve both the micelle and polymersome populations in heterogeneous solutions resulting from irradiation. Although discerning sample composition via DLS requires careful interpretation, these results underscore its efficiency and utility as a screening tool to detect relative changes within a sample.

**Table 1:** Size Comparison Utilizing Three Different Measurement Methods: Cryo-TEM, DLS (Distribution by Number), and DLS (Distribution by Intensity)^a^

	Before irradiation		2 mJ irradiation	
Distribution	Polymersomes	Micelles	Polymersomes	Micelles
DLS (number)	61.83	–	–	12.7 nm
DLS (intensity)	100.2	–	80.9 nm	16.6 nm
Cryo-TEM	48.64	–	50.9 nm	19.5 nm

a Nanopolymersome samples were assembled with 0.035% w/v AuNPs and measured before and after irradiation with 2 mJ Energy.

## Conclusions

This study focused on the development and characterization of a nanoscale polymersome carrier system which demonstrated both enhanced photosensitivity and surfactant resistance, mediated by the concentration of hydrophobic gold nanoparticles within the membrane. It was demonstrated that vesicles could be self-assembled with increasing concentrations of AuNPs in the membrane which increased the mean vesicle size. The AuNP membrane fill percentage was also demonstrated to increase up until a saturation point. AuNP-loaded nanopolymersomes demonstrated an increase in photosensitivity in response to picosecond irradiation congruent with their LSPR with increasing AuNP concentration. Additionally, the laser energy was demonstrated to play a role in membrane disruption, whereby the low energy irradiations resulted in minor alterations at all AuNP concentrations, while the higher energy irradiations resulted in significant membrane disruption with the resulting structural composition of the samples being dependent on the AuNP concentration. In addition to the increase in photosensitivity, the inclusion of DDT-AuNPs was shown to increase vesicle robustness against commonly utilized nonionic and ionic surfactants. At the highest concentration of AuNPs studied, nonionic surfactants required up to 100-fold greater concentration to induce rupture. Interestingly, both ionic surfactants were unable to induce rupture but rather resulted in swelling of the hydrodynamic diameter in all samples prepared with DDT-AuNPs. It can be hypothesized that the inclusion of dodecanethiol-AuNPs increases hydrophobic interactions within the membrane, thus, acting as a scaffold to decrease surfactant susceptibility. Thus, it can be concluded that the inclusion of AuNPs within the hydrophobic region of the polymersome can serve a dual purpose in nanoscale light-responsive carrier systems.

## Supplementary Material



## Notes

Supporting Information: chemical structure of amphiphilic diblock copolymer, histogram of polymersome membrane thickness, knife edge calculation for laser beam width, cryo-TEM image of empty nanopolymersomes, histograms of unirradiated nanopolymersome sizes self-assembled with differing AuNP concentrations, histograms of nanopolymersome sizes self-assembled with differing AuNP concentrations after irradiation, DLS size distribution curves by number and intensity of AuNP-loaded polymersomes in response to the addition of polysorbate 20, Triton X-100, sodium dodecyl sulfate, sodium deoxycholate, and NaCl, DLS size distribution curves by number and intensity of Nile Red-loaded polymersomes in response to the addition of Triton X-100 and sodium dodecyl sulfate, Laurdan fluorescence spectrum, Laurdan generalized polarization ratio as a function of AuNP concentration (PDF)
