# Supplementary material for: Enhanced Photosensitivity and Surfactant Resistance in Nanopolymersome Membranes as a Function of Gold Nanoparticle Incorporation
Source: ACS Appl Opt Mater. 2025 Feb 11;3(2):403–13. doi: 10.1021/acsaom.4c00503 (PMC12333581; doi:10.1021/acsaom.4c00503)
Supplement: Supplementary file 1 [file ot4c00503_si_002.pdf]

Supporting Information

Enhanced photosensitivity and surfactant resistance in nano-polymersome membranes as a function of gold nanoparticle incorporation

Regina L. Salzer[1], Ajay N. Shah[2], Cory J. Trout[2][1], Abby R. Robinson[3], Sujay Ratna[1], Sean M. O'Malley[1,2], Julianne C. Griepenburg[*,1,2]

[1]Center for Computational and Integrative Biology, Rutgers University-Camden, Camden, NJ 08102, USA.
[2]Department of Physics, Rutgers University-Camden, Camden, NJ 08102, USA.
[3]Department of Chemistry, Rutgers University-Camden, Camden, NJ 08102, USA.

*Corresponding author email: j.griepenburg@rutgers.edu

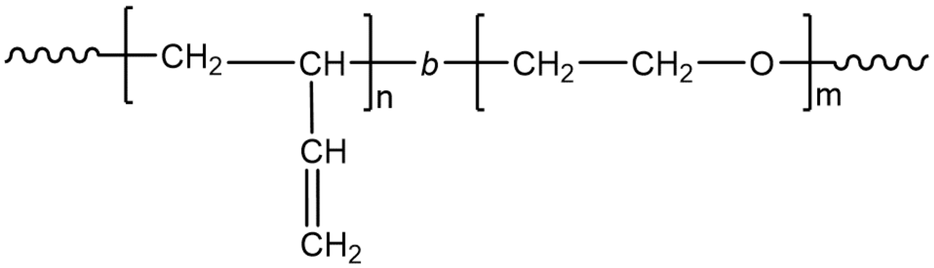

Figure SI-1: The chemical structure of the amphiphilic diblock copolymer (polybutadiene-*b*-polyethylene oxide) used to form polymersomes for the irradiation and surfactant studies outlined in the main text of this work. The specific polymer used had block lengths of n = 33 and m = 20.

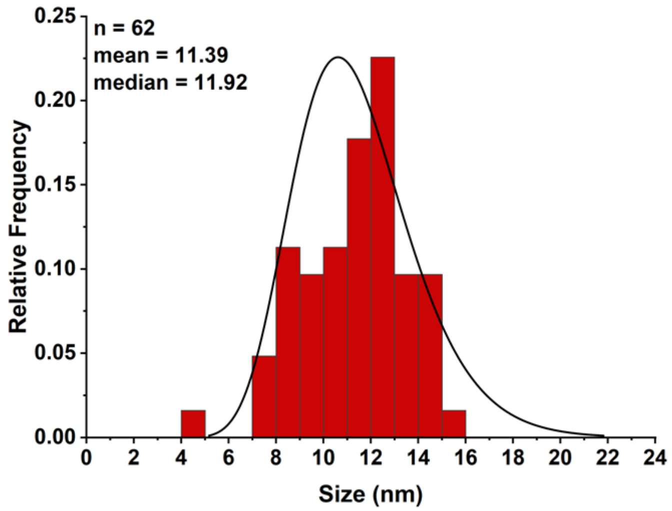

Figure SI-2: A histogram representing the membrane thicknesses of polymersomes assembled without AuNPs as measured from cryo-TEM using ImageJ.

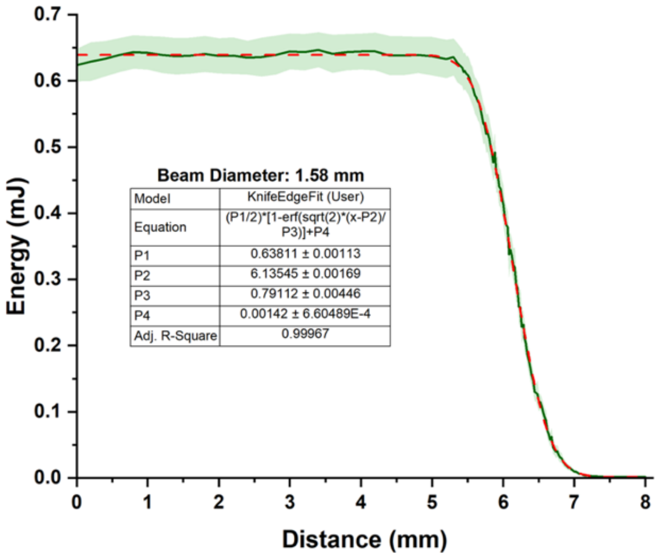

Figure SI-3: The knife edge technique was used to measure the beam width and subsequent spot size of the irradiations performed. The data was fit using the equation shown where parameter P1 is the maximum energy, P2 is the position at which the energy of the beam is half the maximum, P3 is the beam radius, and P4 is the minimum energy.

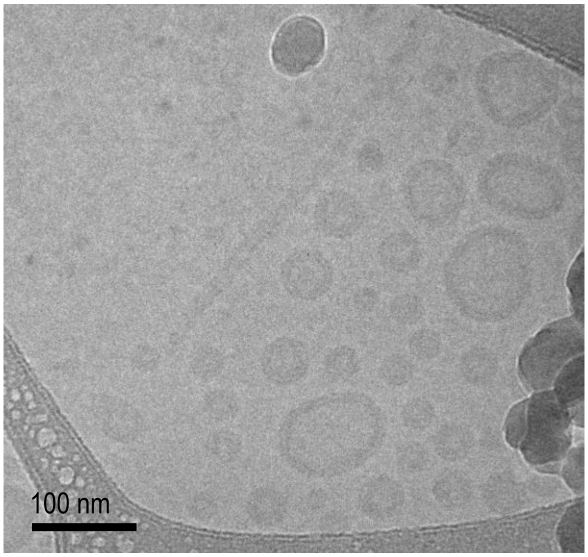

Figure SI-4: Cryo-TEM image of nano-polymersomes self-assembled without AuNPs in the membrane and irradiated with 1.5 mJ energy.

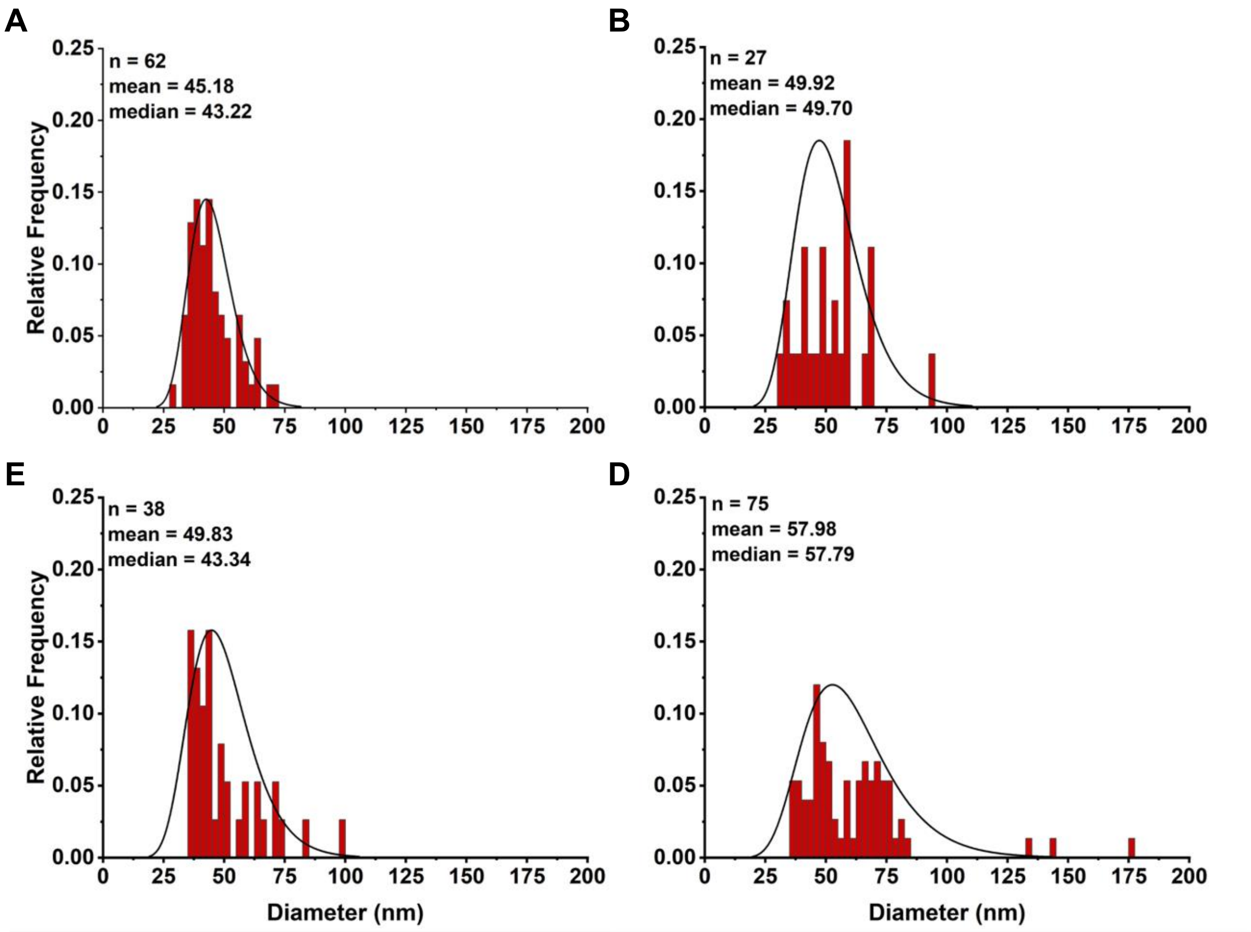

Figure SI-5: Histograms representing sizes of unirradiated nano-polymersomes self-assembled with AuNP concentrations of A) 0% w/v, B) 0.0350% w/v, C) 0.0700% w/v, and D) 0.140% w/v. Vesicles were measured from cryo-TEM images using ImageJ.

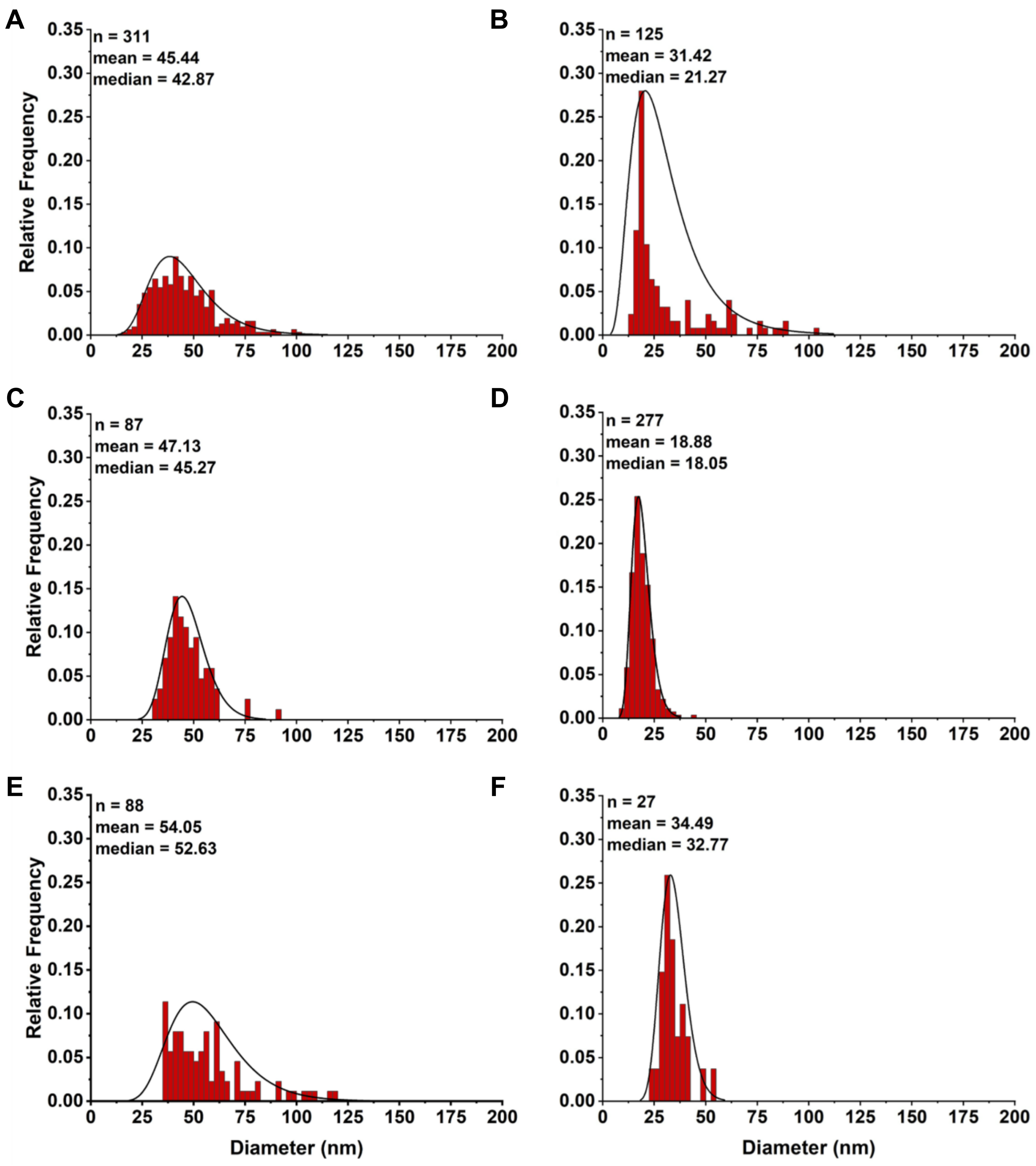

Figure SI-6: Histograms representing sizes of nano-polymersomes irradiated with 0.1 mJ (A, C, E) or 2 mJ (B,D,F) energies, for vesicles self-assembled with AuNP concentrations of A-B) 0.0350% w/v, C-D) 0.0700% w/v, and E,F) 0.140% w/v. Vesicles self-assembled without AuNPs are not shown due to the lack of change upon irradiation. Vesicles were measured from cryo-TEM images using ImageJ.

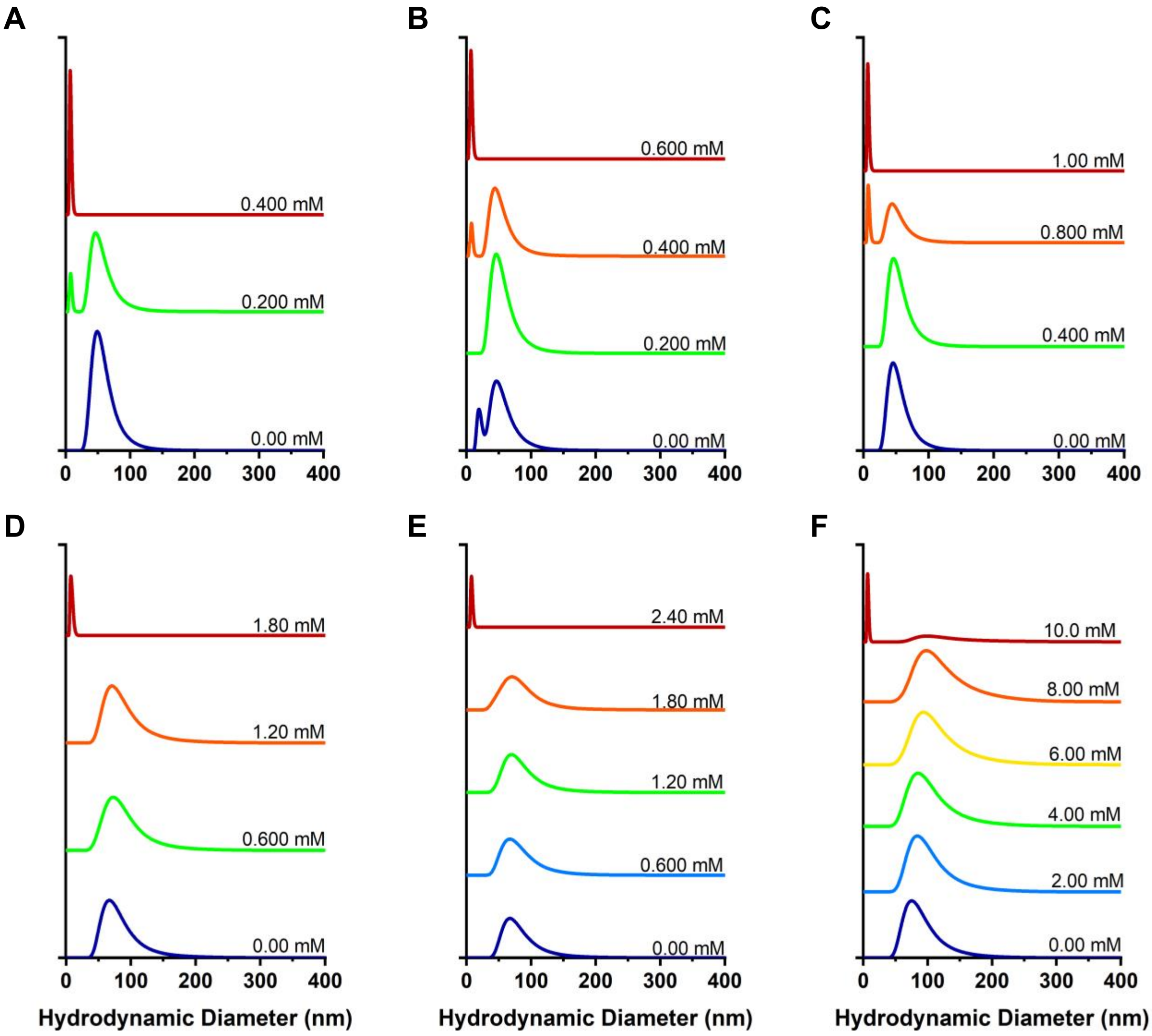

Figure SI-7: DLS size distribution curves by number of nano-polymersomes in response to the addition of polysorbate 20 for vesicles loaded with AuNP concentrations (w/v) of A) 0%, B) 0.0175%, C) 0.0350%, D) 0.0525%, E) 0.0700%, F) 0.140%. The distributions are stacked vertically to allow for visualization of relative changes in hydrodynamic diameter.

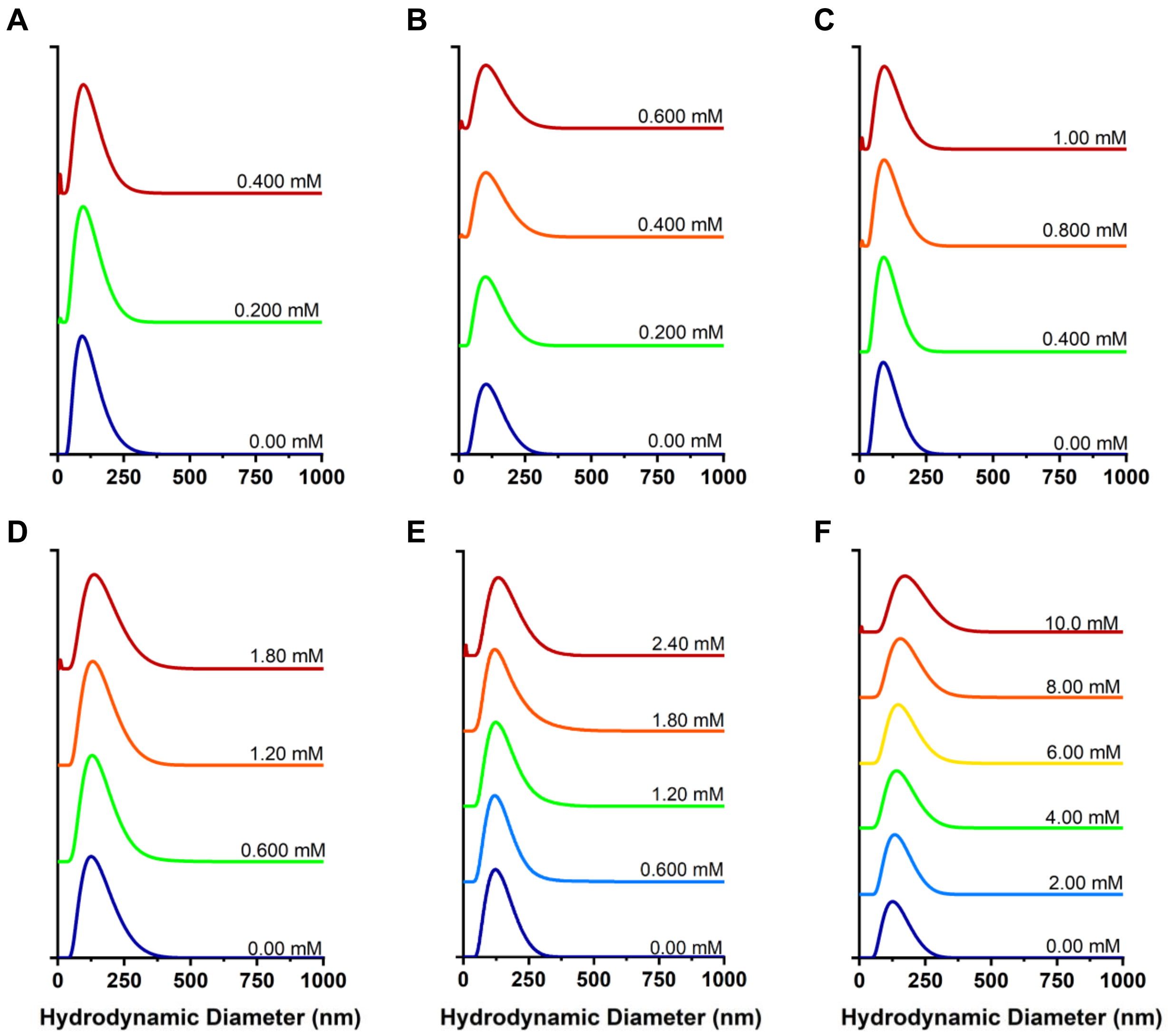

Figure SI-8: DLS size distribution curves by intensity of nano-polymersomes in response to the addition of polysorbate 20 for vesicles loaded with AuNP concentrations (w/v) of A) 0%, B) 0.0175%, C) 0.0350%, D) 0.0525%, E) 0.0700%, F) 0.140%. The distributions are offset and stacked vertically to allow for visualization of relative changes in hydrodynamic diameter.

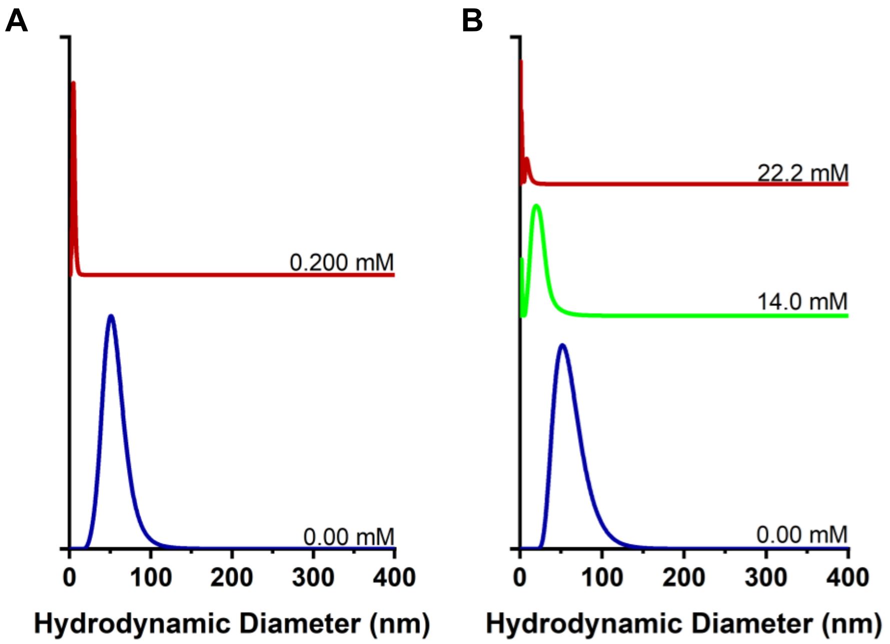

Figure SI-9: DLS size distribution curves by number of nano-polymersomes in response to the addition of Triton X-100 (A) and SDS (B) for vesicles loaded with Nile Red fluorescent dye. The surfactant concentrations were chosen based on the rupture point of empty polymersome samples (Figures SI-11A and SI-14A). The distributions are offset and stacked vertically to allow for visualization of relative changes in hydrodynamic diameter.

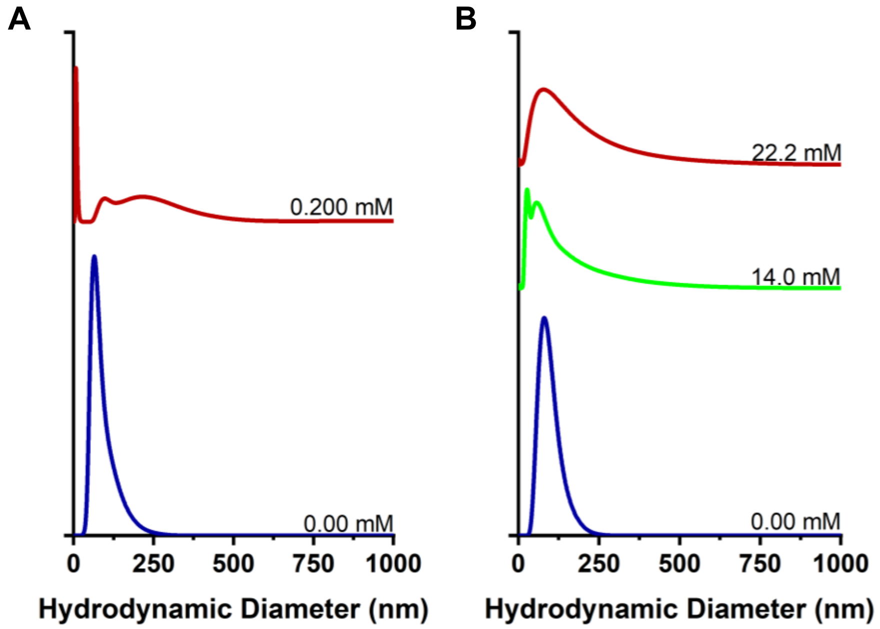

Figure SI-10: DLS size distribution curves by intensity of nano-polymersomes in response to the addition of Triton X-100 (A) and SDS (B) for vesicles loaded with Nile Red fluorescent dye. The surfactant concentrations were chosen based on the rupture point of empty polymersome samples (Figures SI-11A and SI-14A). The distributions are offset and stacked vertically to allow for visualization of relative changes in hydrodynamic diameter.

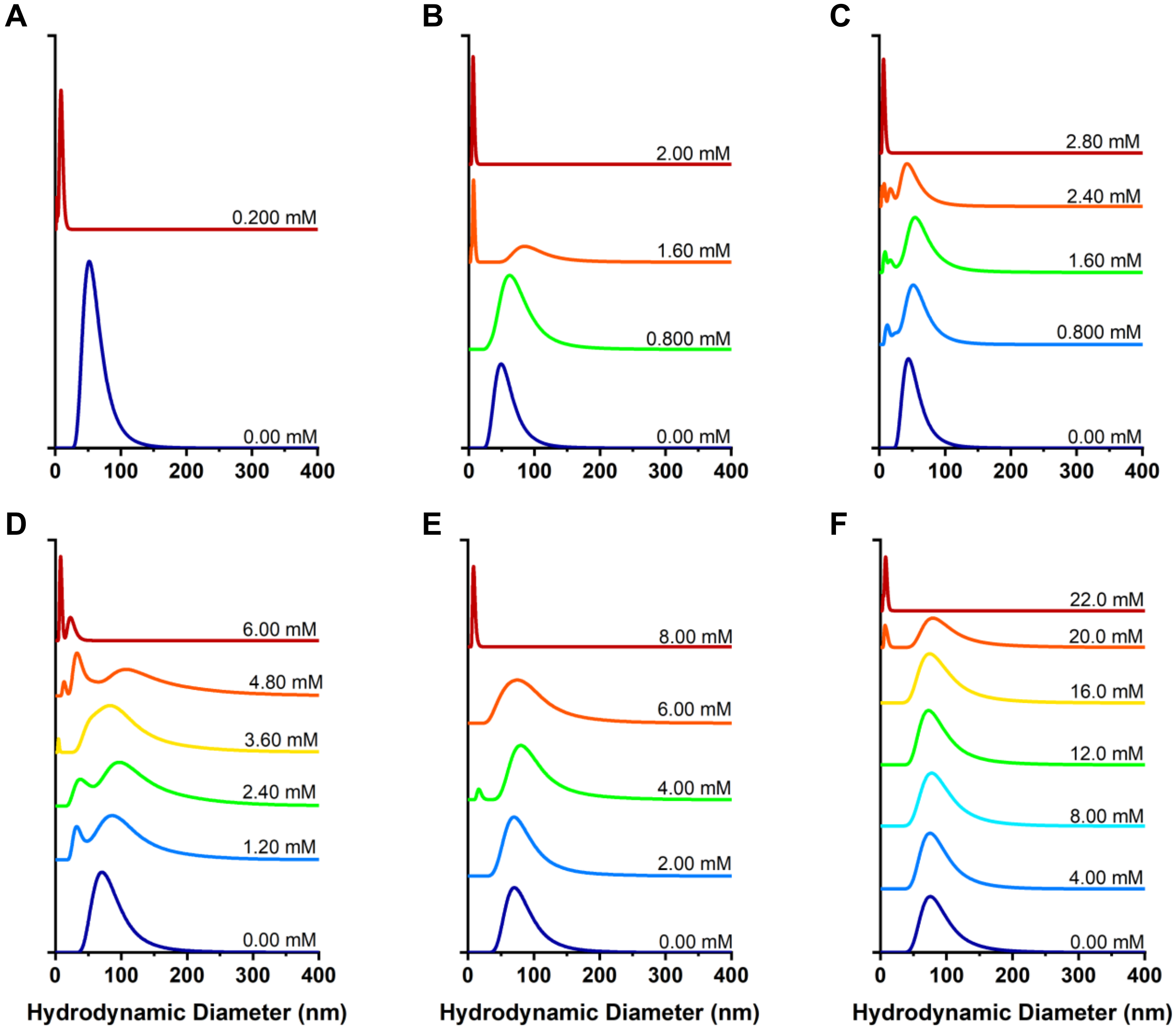

Figure SI-11: DLS size distribution curves by number of nano-polymersomes in response to the addition of Triton X-100 for vesicles loaded with AuNP concentrations (w/v) of A) 0%, B) 0.0175%, C) 0.0350%, D) 0.0525%, E) 0.0700%, F) 0.140%. The distributions are offset and stacked vertically to allow for visualization of relative changes in hydrodynamic diameter.

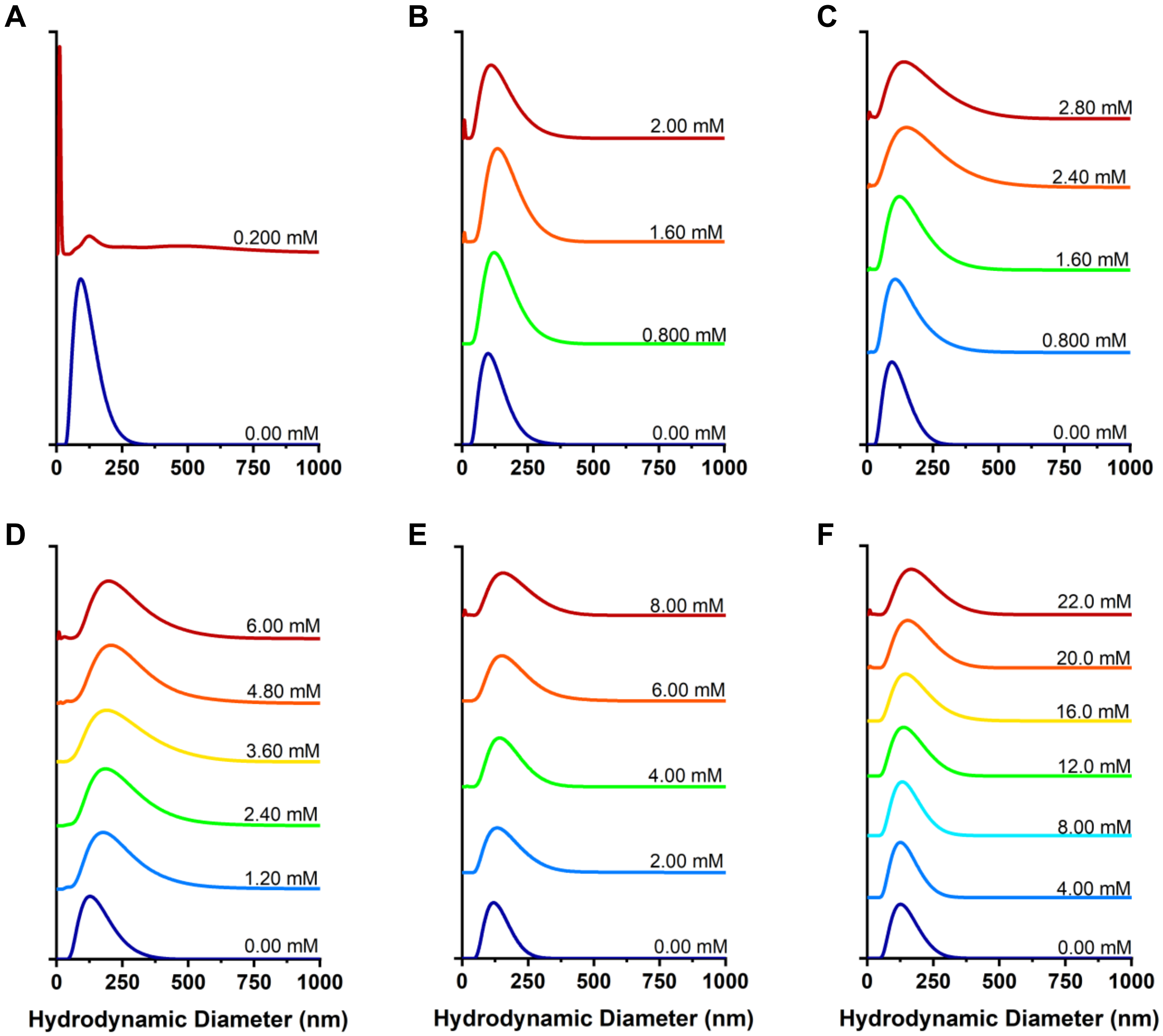

Figure SI-12: DLS size distribution curves by intensity of nano-polymersomes in response to the addition of Triton X-100 for vesicles loaded with AuNP concentrations (w/v) of A) 0%, B) 0.0175%, C) 0.0350%, D) 0.0525%, E) 0.0700%, F) 0.140%. The distributions are offset and stacked vertically to allow for visualization of relative changes in hydrodynamic diameter.

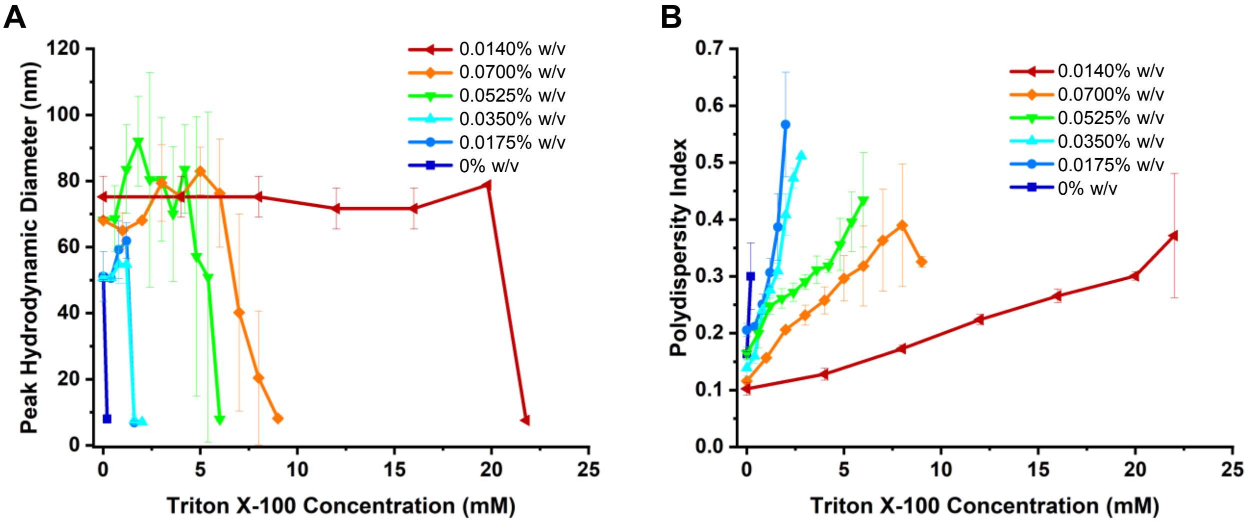

Figure SI-13: A) The peak (mode) hydrodynamic diameter of polymersomes as a function of Triton X-100, as calculated from DLS distributions by number from triplicate studies. The large experimental error noted in the 0.0525% w/v and 0.0700% w/v concentrations are due to one of the three replicates requiring even larger concentrations of surfactant to induce rupture. B) The mean polydispersity index (PDI) as a function of Triton X-100 as reported from DLS measurements. Larger PDI values suggest a broader, more polydisperse sample.

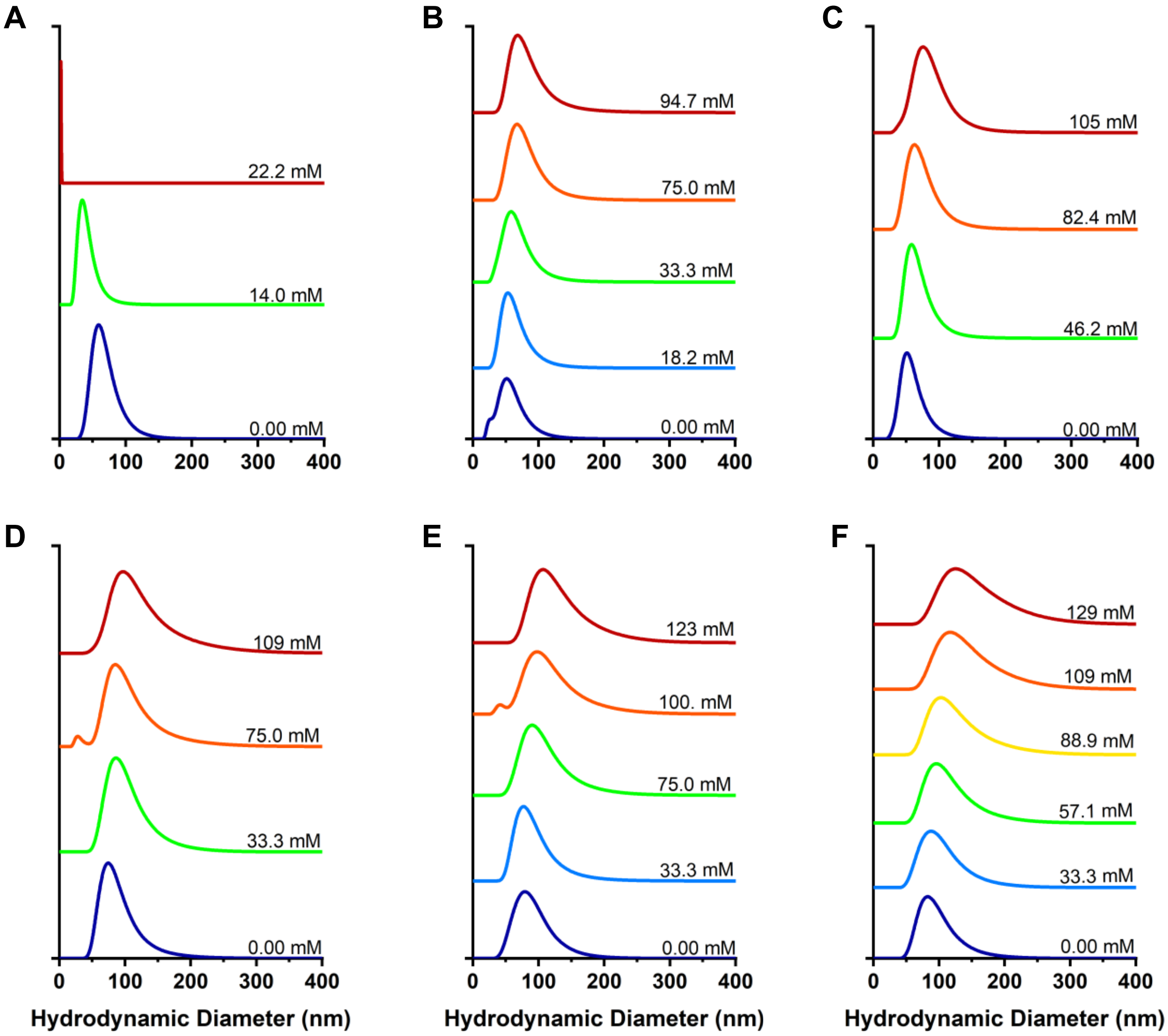

Figure SI-14: DLS size distribution curves by number of nano-polymersomes in response to the addition of sodium dodecyl sulfate (SDS) for vesicles loaded with AuNP concentrations (w/v) of A) 0%, B) 0.0175%, C) 0.0350%, D) 0.0525%, E) 0.0700%, F) 0.140%. The distributions are offset and stacked vertically to allow for visualization of relative changes in hydrodynamic diameter.

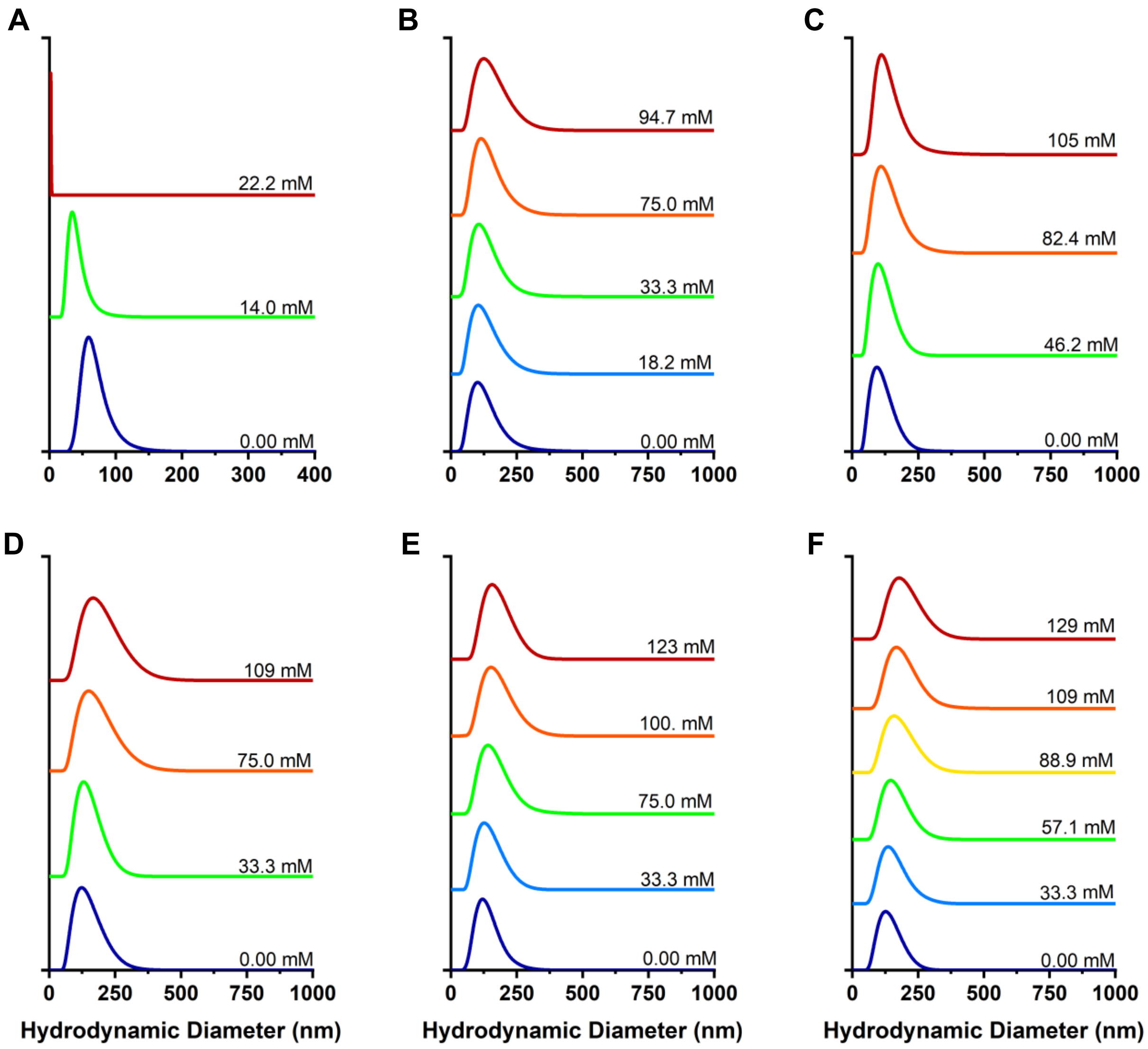

Figure SI-15: DLS size distribution curves by intensity of nano-polymersomes in response to the addition of SDS for vesicles loaded with AuNP concentrations (w/v) of A) 0%, B) 0.0175%, C) 0.0350%, D) 0.0525%, E) 0.0700%, F) 0.140%. The distributions are offset and stacked vertically to allow for visualization of relative changes in hydrodynamic diameter.

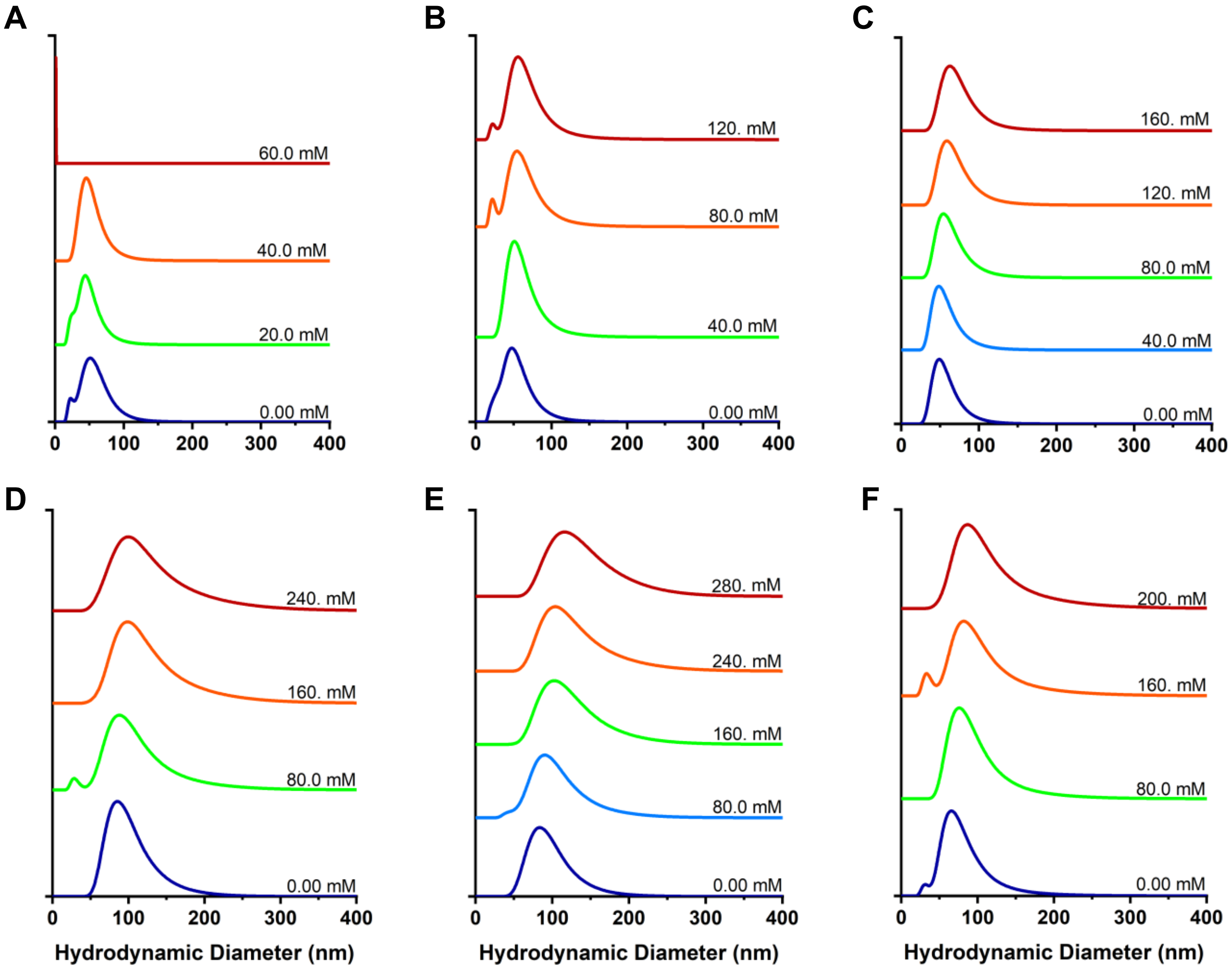

Figure SI-16: DLS size distribution curves by number of nano-polymersomes in response to the addition of sodium deoxycholate for vesicles loaded with AuNP concentrations (w/v) of A) 0%, B) 0.0175%, C) 0.0350%, D) 0.0525%, E) 0.0700%, F) 0.140%. The distributions are offset and stacked vertically to allow for visualization of relative changes in hydrodynamic diameter.

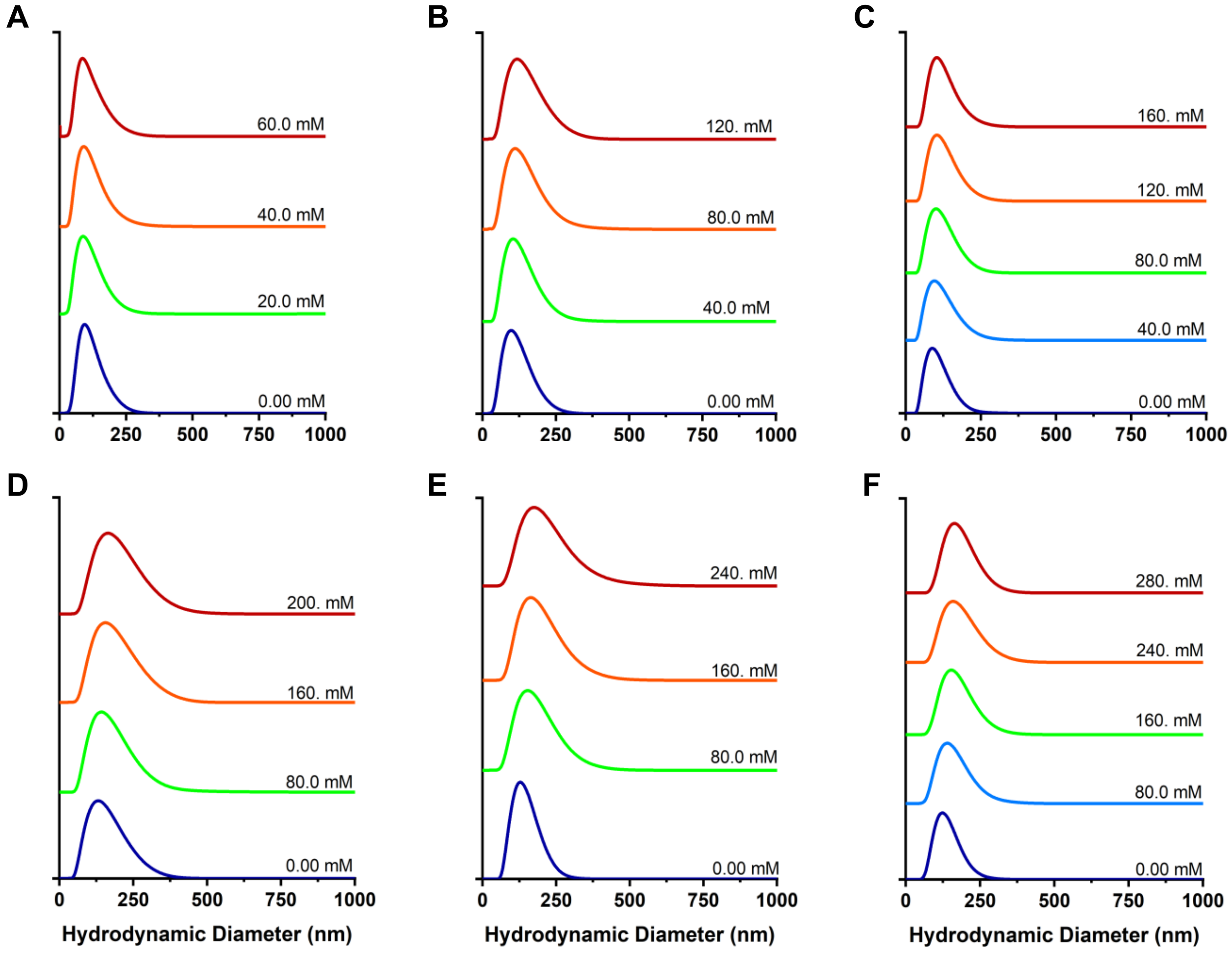

Figure SI-17: DLS size distribution curves by intensity of nano-polymersomes in response to the addition of sodium deoxycholate for vesicles loaded with AuNP concentrations (w/v) of A) 0%, B) 0.0175%, C) 0.0350%, D) 0.0525%, E) 0.0700%, F) 0.140%. The distributions are offset and stacked vertically to allow for visualization of relative changes in hydrodynamic diameter.

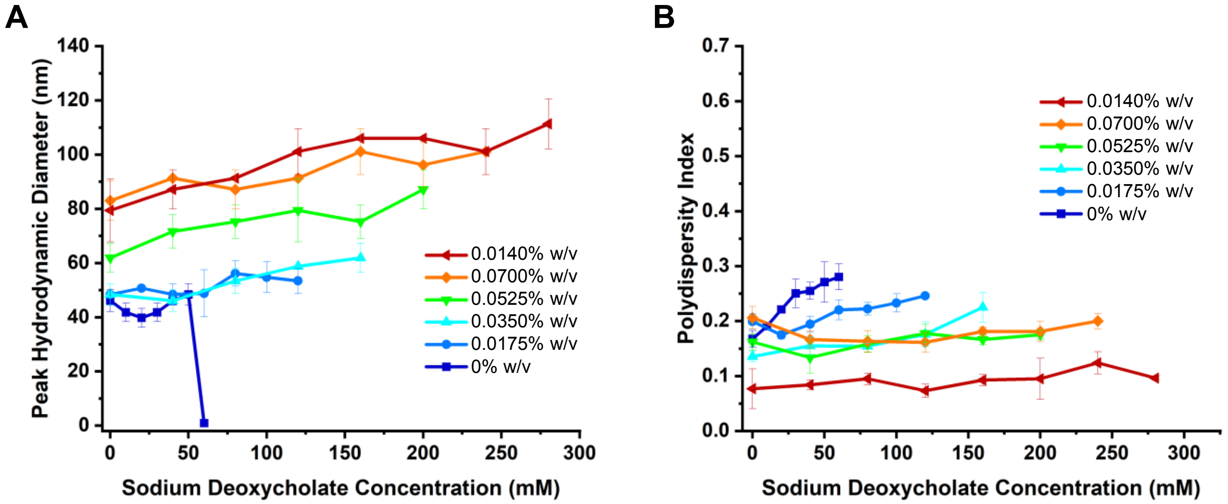

Figure SI-18: A) The peak (mode) hydrodynamic diameter of polymersomes as a function of sodium deoxycholate concentration as calculated from DLS distributions by number from triplicate studies. B) The mean polydispersity index (PDI) as a function of sodium deoxycholate concentration as reported from DLS measurements of the triplicate studies.

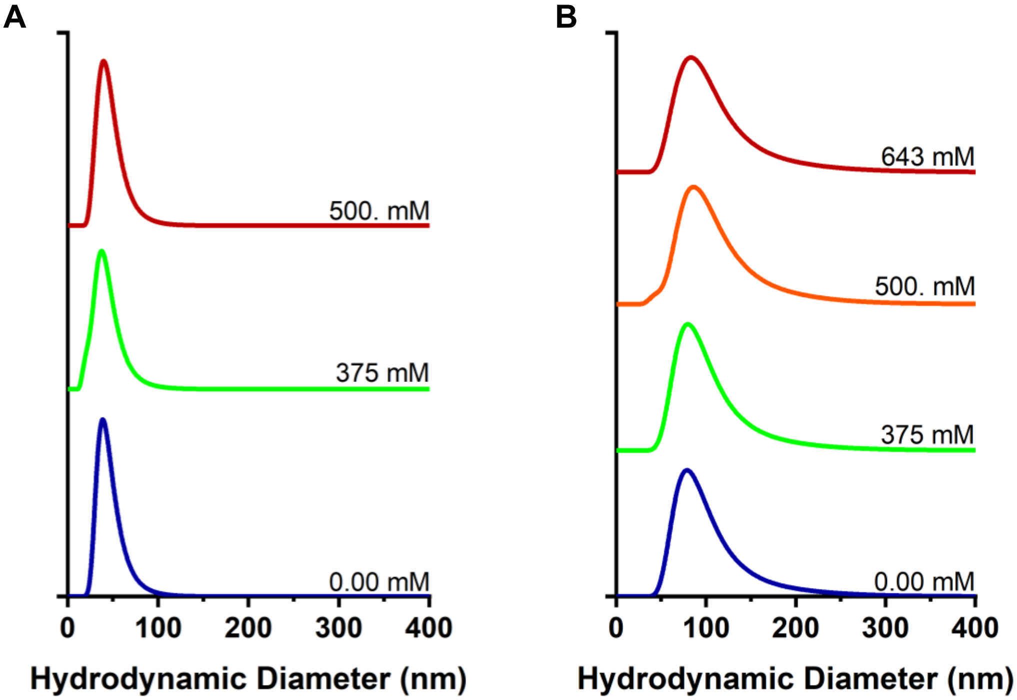

Figure SI-19: DLS size distribution curves by number of nano-polymersomes in response to the addition of NaCl for vesicles loaded with AuNP concentrations (w/v) of A) 0% and B) 0.0700%. The distributions are offset and stacked vertically to allow for visualization of relative changes in hydrodynamic diameter.

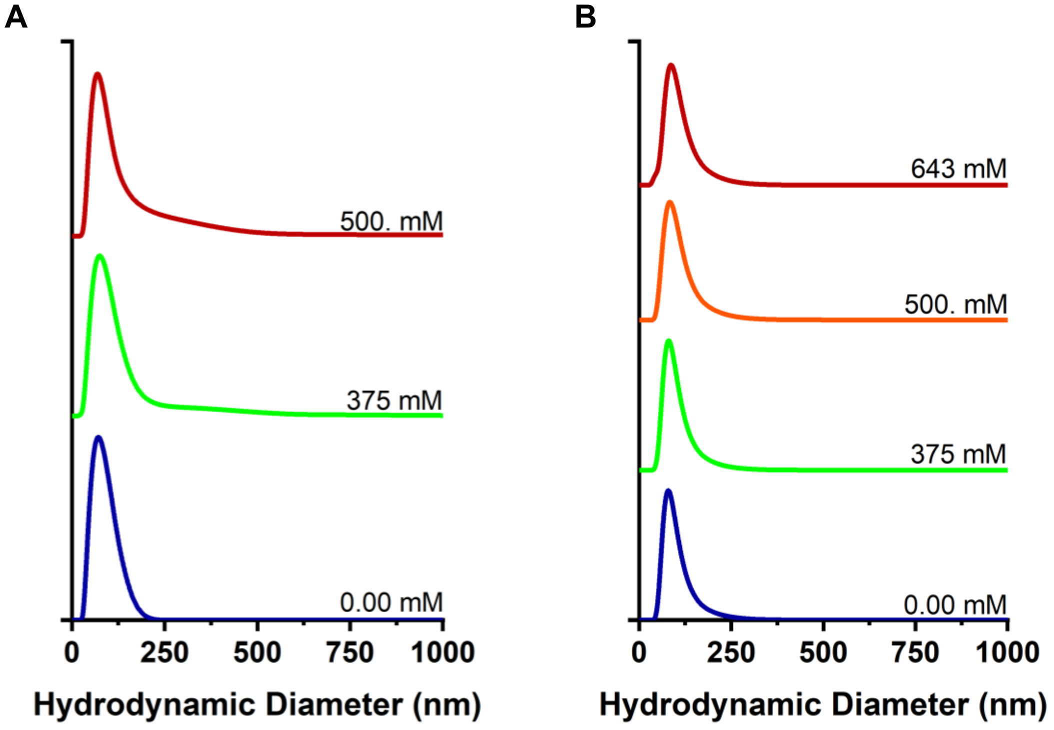

Figure SI-20: DLS size distribution curves by intensity of nano-polymersomes in response to the addition of NaCl for vesicles loaded with AuNP concentrations (w/v) of A) 0% and B) 0.0700%. The distributions are offset and stacked vertically to allow for visualization of relative changes in hydrodynamic diameter.

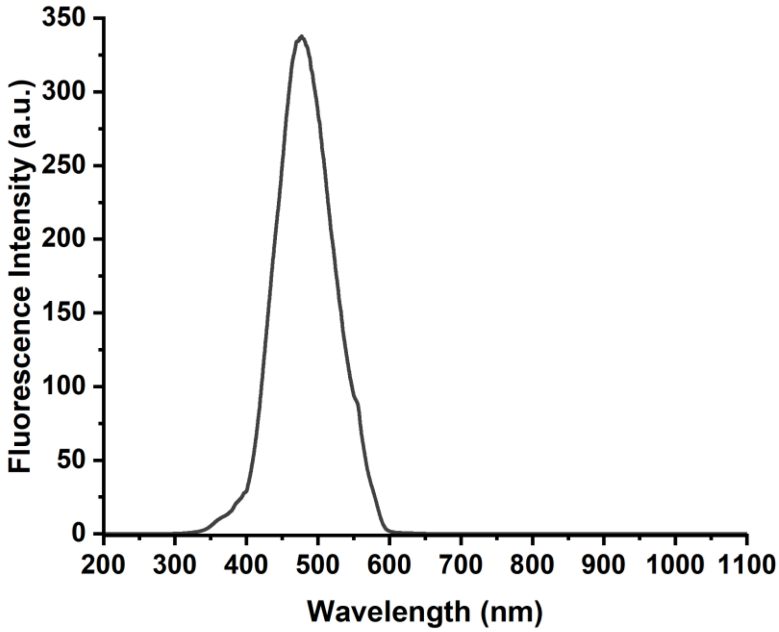

Figure SI-21: A Laurdan fluorescence spectrum for vesicles formed without AuNPs. The intensity values at wavelengths 440 nm and 490 nm were used according to Eq. 2 in the main text to calculate the generalized polarization ratio.

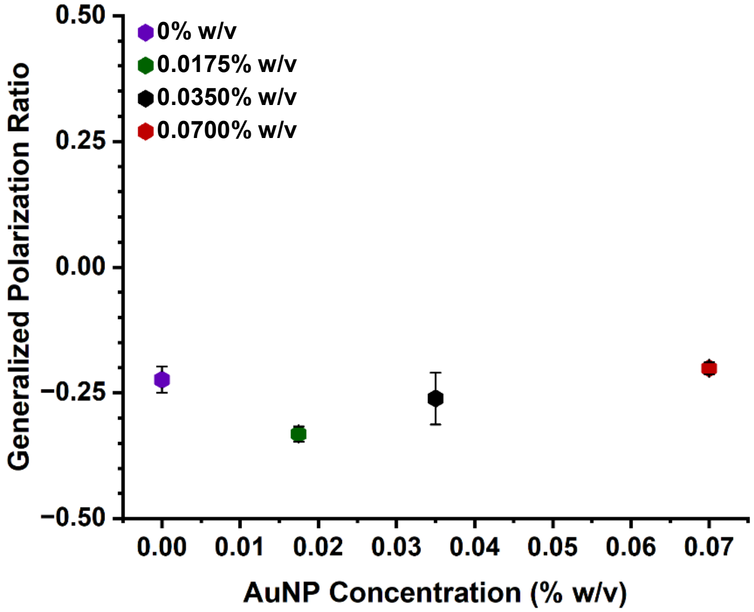

Figure SI-22: The generalized polarization ratio calculated with Eq. 2 in the text using Laurdan fluorescence intensity values for samples containing different concentrations of AuNPs. The 0.140% w/v AuNP concentration was not investigated as the membrane fill percentage was similar to 0.0700% w/v.
